# Super-enhancer hijacking drives ectopic expression of hedgehog pathway ligands in meningiomas

**DOI:** 10.1038/s41467-023-41926-y

**Published:** 2023-10-07

**Authors:** Mark W. Youngblood, Zeynep Erson-Omay, Chang Li, Hinda Najem, Süleyman Coșkun, Evgeniya Tyrtova, Julio D. Montejo, Danielle F. Miyagishima, Tanyeri Barak, Sayoko Nishimura, Akdes Serin Harmancı, Victoria E. Clark, Daniel Duran, Anita Huttner, Timuçin Avşar, Yasar Bayri, Johannes Schramm, Julien Boetto, Matthieu Peyre, Maximilien Riche, Roland Goldbrunner, Nduka Amankulor, Angeliki Louvi, Kaya Bilgüvar, M. Necmettin Pamir, Koray Özduman, Türker Kilic, James R. Knight, Matthias Simon, Craig Horbinski, Michel Kalamarides, Marco Timmer, Amy B. Heimberger, Ketu Mishra-Gorur, Jennifer Moliterno, Katsuhito Yasuno, Murat Günel

**Affiliations:** 1 Yale Program in Brain Tumor Research, Yale School of Medicine, New Haven, CT USA; 2 Department of Neurosurgery, Yale School of Medicine, New Haven, CT USA; 3 Department of Genetics, Yale School of Medicine, New Haven, CT USA; 4 Department of Neurological Surgery, Malnati Brain Tumor Institute of the Robert H. Lurie Comprehensive Cancer Center, Feinberg School of Medicine, Northwestern University, Chicago, IL USA; 5 Department of Neurosurgery, Sun Yat-sen University Cancer Center, 510060 Guangzhou, P. R. China; 6 Department of Biological Sciences, Middle East Technical University, 06800 Ankara, Turkey; 7 Department of Neurosurgery, University of Washington, Seattle, WA USA; 8 Section of Neurosurgery, Dartmouth-Hitchcock Medical Center, Lebanon, NH USA; 9 Department of Neurosurgery, Baylor College of Medicine, Houston, TX 77030 USA; 10 Department of Neurosurgery, Massachusetts General Hospital and Harvard Medical School, Boston, MA 02114 USA; 11 Department of Neurosurgery, University of Mississippi Medical Center, Jackson, MS 39216 USA; 12 Department of Pathology, Yale School of Medicine, New Haven, CT USA; 13 Department of Neurosurgery, Bahcesehir University, School of Medicine, Istanbul, Turkey; 14 Department of Neurosurgery, Marmara University School of Medicine, 34854 Istanbul, Turkey; 15 University of Bonn Medical School, 53105 Bonn, Germany; 16 Department of Neurosurgery, Hopital Pitie-Salpetriere, AP-HP & Sorbonne Université, F-75103 Paris, France; 17 Department of Neurosurgery, Gui de Chauliac Hospital, Montpellier University Medical Center, Montpellier, France; 18 Center for Neurosurgery, University Hospital of Cologne, 50937 Cologne, Germany; 19 Department of Neurosurgery, University of Pennsylvania, Philadelphia, PA USA; 20 Yale Center for Genome Analysis, Yale University West Campus, Orange, CT USA; 21 Department of Medical Genetics Acibadem Mehmet Ali Aydınlar University, School of Medicine, Istanbul, 34848 Turkey; 22 Department of Neurosurgery, Acibadem Mehmet Ali Aydınlar University, School of Medicine, Istanbul, 34848 Turkey; 23 Department of Neurosurgery, Bethel Clinic, University of Bielefeld Medical Center OWL, Bielefeld, Germany; 24 Yale Cancer Center, Yale School of Medicine, New Haven, CT USA

**Keywords:** Cancer genomics, Molecular medicine, CNS cancer

## Abstract

Hedgehog signaling mediates embryologic development of the central nervous system and other tissues and is frequently hijacked by neoplasia to facilitate uncontrolled cellular proliferation. Meningiomas, the most common primary brain tumor, exhibit Hedgehog signaling activation in 6.5% of cases, triggered by recurrent mutations in pathway mediators such as *SMO*. In this study, we find 35.6% of meningiomas that lack previously known drivers acquired various types of somatic structural variations affecting chromosomes 2q35 and 7q36.3. These cases exhibit ectopic expression of Hedgehog ligands, *IHH* and *SHH*, respectively, resulting in Hedgehog signaling activation. Recurrent tandem duplications involving *IHH* permit de novo chromatin interactions between super-enhancers within *DIRC3* and a locus containing *IHH*. Our work expands the landscape of meningioma molecular drivers and demonstrates enhancer hijacking of Hedgehog ligands as a route to activate this pathway  in neoplasia.

## Abstract (web summary)

Hedgehog signalling is known to be linked to oncogenic proliferation. Here, the authors identify structural events as a mechanism of Hedgehog activation in over one-third of driver unknown meningiomas.

## Introduction

Meningiomas are a common form of neoplasia that can arise from any portion of the meninges that cover the brain and spinal cord. The pathologic and anatomic diversity of these lesions results in a wide range of prognoses, and those with aggressive features or falling near critical neurovascular structures can carry high morbidity. Recent studies have identified molecular criteria that stratify risk of recurrence and disease progression^1–5^, however, despite a growing understanding of the need for more aggressive treatment in some patients, surgical resection and radiotherapy remain the sole therapeutic options. Unlike other common tumors, precision medicine approaches and immunotherapies have yet to benefit patients with these lesions^6,7^. Molecular insights pertaining to meningioma pathogenesis are essential for the development of targeted therapeutics with high efficacy and could improve treatment paradigms for patients with these common tumors.

To understand the molecular drivers that underlie meningioma development, previous studies have used sequencing approaches to identify somatic or germline DNA alterations. Biallelic loss of the tumor suppressor *NF2* on chromosome 22q is associated with approximately one-half of all meningiomas^8,9^, and occasionally co-occurs with recurrent mutations in the SWI/SNF subunit *SMARCB1* (ref. ^10^). Among meningiomas with intact *NF2*, recurrent alterations in several well-established neoplasia genes (such as *AKT1*, *PIK3CA*, *SMARCE1*, and *SMO*) account for less than 20% of cases^11–13^, and ongoing clinical trials are investigating the use of relevant precision therapies. Meningiomas harboring recurrent somatic *SMO* mutations are part of a larger group of samples that exhibit activation of the Hedgehog (Hh) signaling pathway, which also includes cases with somatic biallelic loss of *SUFU* or *PRKAR1A*^*A17D*^ mutation^14^. Interestingly, studies have also identified somatic mutations in genes previously not associated with cancer, including the ubiquitin ligase *TRAF7*, pluripotency factor *KLF4*, and catalytic subunit of RNA polymerase II, *POLR2A*^11,14^. Despite this considerable progress, the genomic pathogenesis of over one-fifth of meningiomas remains obscure, preventing the design and utilization of rational precision therapies for these patients. As previous exome-sequencing studies have failed to identify pathogenic coding alterations in these mutation-negative meningiomas, we hypothesized that non-coding genomic events may play a role in their pathogenesis.

Meningiomas with Hh pathway activating mutations comprise approximately 6.5% of all cases^14^. This pathway plays an essential role in embryonic development and is regulated in a dose-dependent manner via Indian, Sonic, and Desert Hedgehog ligand expression. Binding of these ligands to the receptor Patched1 (PTCH1) causes released inhibition of Smoothened (SMO), resulting in downstream pathway activation via GLI family transcription factors^15^. Changes in physiologic expression levels are sufficient to drive dramatic clinical phenotypes, such as the association of dysregulated *IHH* with craniosynostosis, brachydactyly, and acrocallosal syndromes^16–19^. In these developmental disorders, genomic rearrangements that disrupt normal transcriptional regulation of this locus have been implicated, resulting in constitutive gene expression and Hh pathway activation^16–19^. Increased expression of *IHH* has also been identified in malignant processes, including digestive tract, prostate, and colorectal cancers^20–22^, and may also occur in meningiomas^23^. However, the molecular mechanisms that lead to increased *IHH* expression in neoplasia have not been established. Unraveling these processes may elucidate important routes to oncogenesis in tumors that lack established genomic drivers, particularly those that exhibit Hh pathway activation.

In this study, we perform genomic and epigenomic analyses to investigate pathologic drivers of meningiomas that lack well-established somatic alterations. A mutual exclusivity analysis using whole-exome sequencing (WES) data identifies somatic copy number alterations (SCNAs) as candidate drivers of meningiomas, including those on chromosomes 2q, 3p, and 7q, as well as multiple whole-chromosomal gains that occur in individual samples. Whole-genome sequencing (WGS) identifies diverse structural variations (SVs) affecting 2q35 and 7q36.3, for which RNA-sequencing (RNA-Seq) analysis demonstrates association with ectopic expression of the Hh ligands *IHH* and *SHH*, respectively. We show that these samples exhibit activation of the Hh signaling pathway, similar to meningiomas with recurrent *SMO* mutations, using RNA-Seq and multiplexed immunofluorescence experiments. To gain mechanistic insight into the relationship between the structural variations and ectopic expression of *IHH*, we perform genome-wide chromosome conformation capture (Hi-C) followed by H3K27ac ChIP-seq (HiChIP) analysis for chromatin interactions of meningiomas, comparing those with and without recurrent *IHH* tandem duplications, as well as H3K27ac ChIP-seq analysis for super-enhancer identification. We discover de novo chromatin interactions involving super-enhancers within *DIRC3* and a locus containing *IHH* in the samples with tandem duplication. We thus identify super-enhancer hijacking of Hh ligands as a route to Hh signaling activation in meningiomas.

## Results

### Mutual exclusivity analysis identifies SCNA drivers of meningiomas

**Fig. 1:**
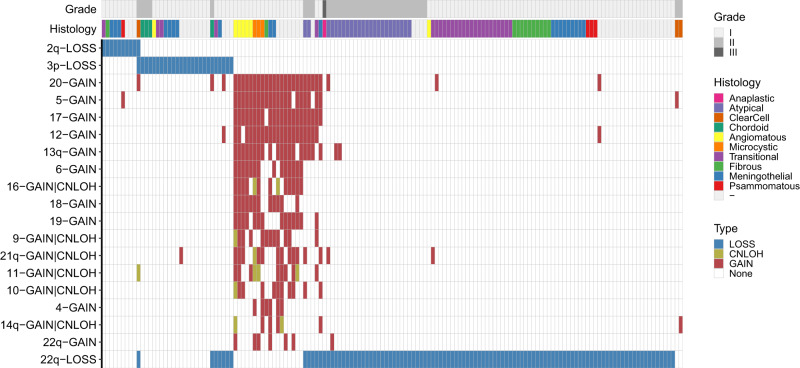
Large SCNAs mutually exclusive to 22q-loss. Meningiomas lacking alterations in known genomic drivers, including loss of chromosome 22q, frequently exhibited somatic copy number losses in chromosomes 2q or 3p, or co-occurring whole-chromosomal gains or copy-neutral loss of heterozygosity (CN-LOH) of 16 additional chromosomes. Meningioma samples that acquired at least one of the events that are mutually exclusive to 22q-loss or 22q-loss (*n* = 150) are aligned horizontally while chromosomes (or chromosome arms) with SCNA types are vertically aligned. Only the events that are mutually exclusive to 22q-loss (as well as 22q-loss itself) are presented. If a chromosome name does not contain p or q arm designation such as 20-GAIN, the event covered at least 50% of both arms. Most of such gains covered >80% of the chromosome (or the arm for acrocentric chromosomes). SCNA types are colored according to the legend at the bottom right (Type). The WHO grade and histology of the samples are labeled using color bars on top. Source data are provided as a Source Data file.

To understand genomic alterations underlying the oncogenesis of mutation-negative meningiomas, we performed WES of 293 tumor-normal pairs, among which 251 demonstrated sufficient quality of somatic copy number profiles for statistical analysis (Methods, Supplementary Data 1). Hypothesizing that genomic structural events may drive oncogenesis among mutation-negative cases, we began by analyzing large SCNAs. Based on the distribution of SCNA sizes among our cohort, we defined ‘large’ SCNAs as those covering at least nine megabases (Mb) of a chromosomal arm collectively (Methods, Supplementary Fig. 1 and Supplementary Data 2). We identified 18 SCNAs that were statistically mutually exclusive (at a 5% false discovery rate [FDR]) with losses of chromosome 22q that overlap with *NF2* (22q-loss), a well-established driver of meningiomas (Fig. 1, Supplementary Fig. 2 and Supplementary Data 3). They consisted of 16 co-occurring gains (including copy-neutral loss of heterozygosity [CN-LOH], Supplementary Data 4), chromosome 3p loss (3p-loss) and chromosome 2q loss (2q-loss).

Among the co-occurring copy number gains or CN-LOHs, a vast majority (96.7%, 234 of 242) were found to be whole-chromosomal events that covered 80% or more of the chromosome (Supplementary Data 4). As the distribution of the number of whole-chromosomal gains acquired by each sample demonstrated clear stratification (Supplementary Fig. 3), we classified samples acquiring four or more such events as belonging to a unique genomic subgroup, which was found to comprise the majority of angiomatous and microcystic meningiomas as previously described^24–26^. Meningiomas with loss of chromosome 3p have recently been reported in higher-grade aggressive lesions, and this event tends to co-occur with damaging mutations in *BAP1* or *PBRM1* (refs. ^27,28^). In our case, however, more than 80% of the samples that acquired 3p-loss (that overlapped *BAP1* and *PBRM1*) without 22q-loss were grade I (Supplementary Data 5), extending a role for this driver to a broader spectrum of meningiomas that includes low grade lesions. It is notable that most of these tumors also acquired chromosome 1p loss (88.2%, Supplementary Data 5) while they were mutually exclusive with 14q-loss (0% co-occurred, *q*-value = 0.0031, Supplementary Data 3).

**Fig. 2:**
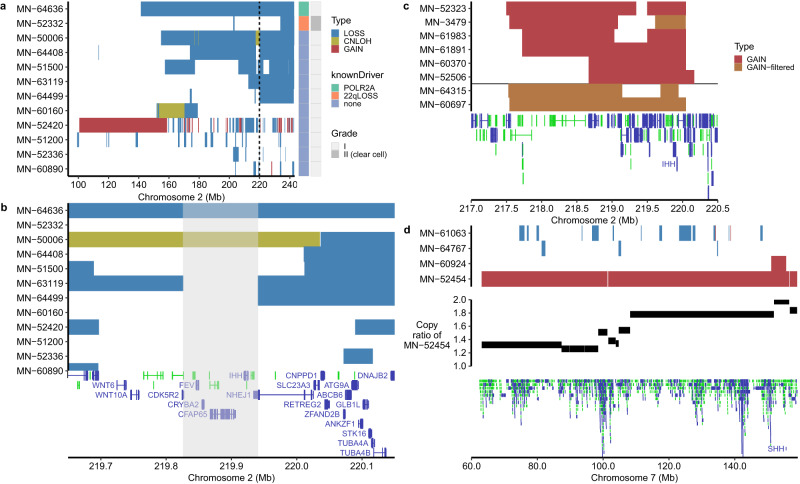
SCNAs affecting 2q and 7q suggest the involvement of *IHH* and *SHH* in meningioma oncogenesis. **a** Distribution of SCNAs across the entire chromosome 2q arm for samples that acquired at least one somatic copy number loss on chromosome 2q are shown. Aside from a single case with *POLR2A* recurrent mutation (MN-64636), all tumors exhibited complex rearrangements. Event types are colored according to the legend at the top right (Type). Two dashed lines mark a 500 kilobases (kb) region from 219.65 Mb to 220.15 Mb, which will be shown in (**b**). The known driver alteration and WHO grade for the respective samples are shown at the right of the main panel. **b** The 500 kb interval, highlighted in (**a**), is extracted to provide a detailed view. Among the samples with complex rearrangements, we observed consistent copy number neutrality of a small genomic region that encompassed *IHH* and three other genes, represented by a gray-shaded area. The colors of SCNA types are the same as (**a**). At the bottom, Gencode basic genes (v38lift37) in this region are shown (protein coding genes in blue; used also in (**c**, **d**). For each gene, only the canonical transcript is selected. **c** A cluster of focal gains at 2q35 found in the remaining mutation-negative meningiomas is shown. An interval from 217.0 Mb to 220.5 Mb on chromosome 2 is depicted. In all these cases, *IHH* was amplified. The bottom two samples as well as a focal gain of MN-3479 were identified after applying weaker filtering conditions to copy number gain calls by ExomeCNV (GAIN-filtered, shown in brown color, see Methods). **d** Four of the remaining mutation-negative samples were found to harbor SCNAs on chromosome 7q arm, which encompasses the gene *SHH*. These SNCAs included chromothripsis, a focal gain at 7q36.3 and complex gains. The middle panel shows the copy ratio (tumor vs. normal) variation across 7q arm for MN-52454. This plot shows the whole chromosome 7q arm. Source data are provided as a Source Data file.

The final mutually exclusive somatic SCNA was partial loss of chromosome 2q, which has not been reported to be associated with meningiomas previously. Interestingly, except for a single case that acquired a known driver mutation in *POLR2A* and simple large 2q-loss, all other meningiomas (*n* = 9) exhibited complex rearrangements, including cases of possible chromothripsis (Fig. 2a), and a remarkable property of preserving or regaining copy neutrality for a short segment of 115 kilobases (kb, from 219,825,716 to 219,940,962, Fig. 2b). Four protein-coding genes localize to this region (*FEV*, *CRYBA2*, *CFAP65* and *IHH*), one of which is the secreted hedgehog (Hh) pathway ligand, *IHH*. We found two more meningiomas that showed chromothripsis but covering only ~4 Mb of 2q arm collectively and exhibited the same property (Fig. 2a, bottom two samples). Thus, a total of 11 meningiomas acquired complex rearrangements on 2q arm without losing *IHH*, and all but one of those were primary and pathologically grade I (Fig. 2b and Supplementary Data 6). One exceptional case was an irradiated and recurrent meningioma with clear cell histology that acquired multiple driver alterations and losses of chromosomes 3p and 22q in addition to complex rearrangements of 2q (Fig. 1). In contrast to known Hh meningiomas (those with a *SMO*, *SUFU* or *PRKAR1A* mutation), which are typically found at the midline anterior skull base, 70% (7 of 10) of the primary meningiomas in this subgroup originated from non-skull base locations, and two of the three skull-base meningiomas were found in lateral regions (Supplementary Data 6). We did not observe any other chromosome arms that were statistically mutually exclusive to 22q-loss and exhibited this unique property as the events on 2q (however, see below).

After excluding samples that acquired known driver alterations as well as those that acquired SCNAs described above, we analyzed the remaining mutation-negative samples (*n* = 52) by including shorter SCNAs (total length > 1 Mb). We found that 19 of these samples acquired at least one such a SCNA. Notably, we identified a cluster of focal gains at 2q35 (*n* = 6, Fig. 2c and Supplementary Data 7), occurring at an almost identical region of 2.68 Mb (217.49 Mb to 220.17 Mb) that contained *IHH*. We found two additional tumors that likely acquired similar focal gains by using a weaker filtering condition (Methods, bottom two samples of Fig. 2c). These meningiomas were all primary and typically grade I tumors (7 of 8), and unlike samples with 2q complex re-arrangements, they all originated from the skull base, typically from medial locations (6 of 8; Supplementary Data 8).

Interestingly, our analysis also identified four meningiomas that acquired events on chromosome 7q (Supplementary Data 9). Two of these exhibited chromothripsis, while one showed a focal gain at 7q36.3 that involved *SHH* and another exhibited a complex pattern of gains among which the highest copy number segment contained *SHH* (Fig. 2d). The three tumors with either 7q chromothripsis or focal gain at 7q36.3 were grade I and originated from the skull base, while the final sample with complex gains was grade I and originated from outside the skull base (Supplementary Data 10). Among the remaining mutation-negative meningiomas, we did not identify chromosomal arms affecting more than one sample.

### WGS identifies SVs with breakpoints near *IHH* and *SHH*

As the SCNAs on 2q and 7q suggested the involvement of SVs in meningioma oncogenesis, we performed SV analysis using WGS of mutation-negative meningiomas, including some samples that acquired the SCNAs described above (Supplementary Data 11). We confirmed tandem duplications from four meningiomas with focal gains at 2q35, as well as chromothripsis and other complex events on 2q and 7q (Table 1 and Supplementary Fig. 4). Furthermore, we identified two inter-chromosomal translocations with a 2q35 breakpoint at 220,029,008 and 220,034,453 (MN-52288 and MN-63401, respectively). These breakpoints, as well as one of the breakpoints of 2q-chromthripsis (MN-52420, at 220,090,023) and 2q complex rearrangements (MN-51500, at 220,073,582) were mapped at proximity to those of tandem duplications (220,056,209-220,067,681), revealing a hotspot of breakpoints (Table 1). Additionally, we identified an inversion and an inter-chromosomal translocation with a breakpoint falling between *SHH* and *LMBR1* at 7q36.3, suggesting the disruption of linkage between *SHH* and a long-range regulatory element in an intron of *LMBR1* (ref. ^29^), with this event most likely resulting in transcriptional dysregulation (discussed below).

**Table 1:** Structural variations identified from WGS analysis

Samples	Structural variation^a^	Description	WES SCNA	Coverage^b^ (Tumor/Blood)	Sex	Grade	Histology	Location^c^
Events with a breakpoint between *IHH* and *ABCB6*								
MN-60370	2:217,875,406-220,056,209	Tandem duplication	Focal gain at 2q35	43.7/18.9	Female	I	Transitional	SB
MN-61891	2:218,024,502-220,058,113	Tandem duplication	Focal gain at 2q35	80/35.5	Female	I	Transitional	SB
MN-61983	2:218,258,592-220,067,681	Tandem duplication	Focal gain at 2q35	15.9/.	Male	I	Meningothelial	SB
MN-52323	2:218,152,515-220,060,111	Tandem duplication	Focal gain at 2q35	80.8/48	Male	I	Meningothelial	SB
MN-52420	2:161,061,851-219,931,428 (DUP), 2:220,090,023-242,064,976 (INV)	Tandem duplication and inversion, a part of 2q chromothripsis	2q chromothripsis	79.8/31.5	Male	I	Meningothelial	NSB
MN-51500	2:218,324,785-220,073,582	Inversion between *DIRC3* and *ZFAND2B*	2q35 complex rearrangement	12.9/.	Male	I	–	NSB
MN-52288	2:pter-220,029,008||10:77,508,708-qter, 10:pter-77,508,707||2:220,029,009-qter	Reciprocal inter-chromosomal translocation (*SLC23A3*-*LRMDA* fusion)	None	79.5/30.7	Female	I	Meningothelial	SB
MN-63401	2:pter-220,034,453||1:218,632,910-qter, 15:pter-66,094,810||2:220,034,456-qter	Inter-chromosomal translocation (*SLC23A3*-1q41/15q22.31)	No WES	67.8/.	Female	I	Meningothelial	SB
Events with a breakpoint between *SHH* and *LMBR1*								
MN-52454	7:106,249,821-156,213,808	Tandem duplication, a part of 7q chromothripsis	7q complex gains	61.1/26.2	Female	I	-	NSB
MN-61063	7:103,724,817-155,637,947 (DEL), 7:134,233,909-155,637,315 (DUP)	Deletion and tandem duplication, a part of 7q chromothripsis	7q chromothripsis	70.5/37.5	Male	I	Meningothelial	SB
MN-52406	7:15,845,930-156,073,765	Inversion	None	67.9/37.6	Female	I	Meningothelial	SB
MN-63565	7:pter-156,288,487||8:69,296,588-qter, 8:pter-69,296,287||7:156,288,488-qter	Reciprocal inter-chromosomal translocation (7q36.3-*C8orf34*)	None	56.2/19	Male	I	Meningothelial	SB
MN-61486^d^	7:pter-155,619,365||18:39,033,373-qter	Inter-chromosomal translocation (7q36.3-18q12.3)	None	13.1/.	Male	I	Meningothelial	SB
Other events								
MN-50008	2:219,483,604-219,925,168	Inversion between *IHH* and *PLCD4* (IHH is disrupted)^e^	None	61.4/37.5	Male	I	Meningothelial	SB
MN-52396	12:51,635,001-63,918,911	Tandem duplication, a part of 12q chromothripsis	No WES	67/36.7	Female	II	Atypical	NSB
MN-52391	1:pter-156,104,233||19:45,974,291-qter, 19:pter-45,974,314||1:156,104,245-qter	Reciprocal inter-chromosomal translocation (*FOSB*-*LMNA* fusion)	None	61.8/35.4	Female	I	Transitional	NSB
MN-62105	22:pter-29,683,454||2:208,438,909-qter, 2:pter-208,439,319||22:29,683,029-qter	Reciprocal inter-chromosomal translocation (*EWSR1*-*CREB1* fusion)	None	56.9/17.8	Female	I	Meningothelial	SB

All the events identified by MANTA from our samples (*n* = 24) can be found in Supplementary Data 11. Among the 24 samples, 19 acquired at least one structural variation (SV). Two of the 19 samples (MN-60690 and MN-61306) not listed here were subjected to tumor-only low-coverage WGS and their targeted SVs (tandem duplication at 2q35 implicated from WES and an event near IHH implicated from RNA-Seq) were not detected.

^a^Structural variations were described using a (chromosome):(start)-(end) format for duplication and inversion while a (the first chromosomal segment)||(the second chromosomal segment) format for inter-chromosomal translocations.

^b^The mean coverages of tumor and blood samples. The missing value was indicated by a dot.

^c^SB, skull base. NSB, non-skull base.

^d^This sample was sequenced to follow-up the finding of the ectopic expression of *SHH*.

^e^This event is unlikely to be a driver alteration (see the main text).

We identified two additional interesting cases that involved genes involved in Hh signaling. In the first one, we identified a reciprocal inversion (2:219,483,604-219,925,175) that disrupted *IHH*, as one of the breakpoints fell within the first exon of this gene (MN-50008). This ruled out ligand activation as the mechanism underlying meningioma formation, and we notably observed a splice acceptor mutation (ENST00000593685.1:c.955-1G>C) in *DYRK1B*. *DYRK1B* was previously established as a negative regulator of Hh signaling, with a loss of this gene resulting in increases in expression of Hh pathway target genes and effectors^30,31^. In a second sample, we identified a tandem duplication involving *GLI1* (MN-52396, 12:51,635,001-63,918,911) accompanied by complex rearrangements (Supplementary Fig. 4 and Table 1). As the *GLI1* transcription factor is a primary effector of the Hh signaling pathway, amplification of this gene most likely underlies Hh activation in this meningioma.

Finally, we also identified two gene fusions, *LMNA*-*FOSB*, t(1;19)(q22;q13), and *EWSR1*-*CREB1* (t(2;22)(q34;q12), Table 1). The former is listed among the supplemental candidate events of a previously reported meningioma sample^12^. The latter event has been found in multiple other neoplasia, including diverse sarcomas and carcinomas^32^. Future studies are needed to understand potential contributions of these fusion events to meningioma formation.

### SVs result in ectopic expression of Hh ligands and pathway activation

**Fig. 3:**
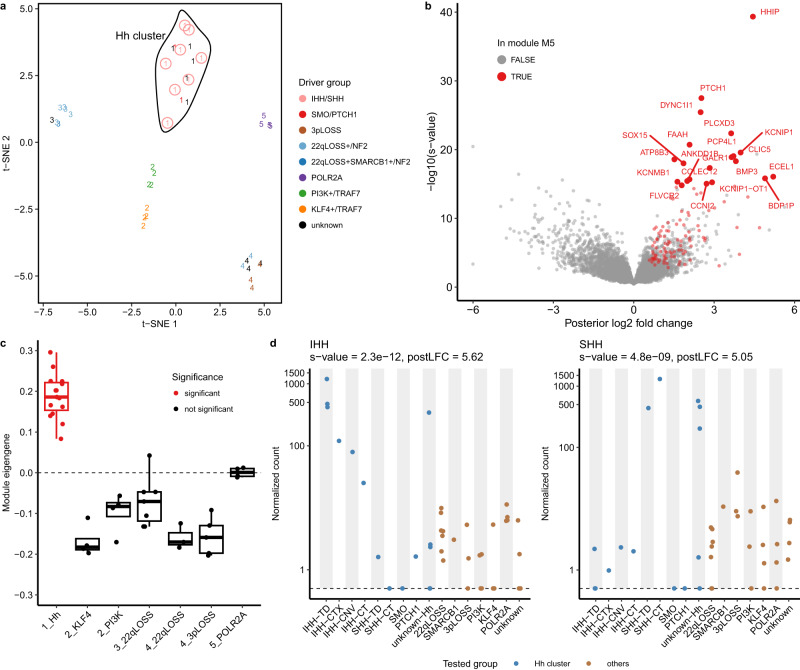
RNA-Seq data supports Hh signaling activation in meningiomas acquiring SVs on 2q35 and 7q36.3. **a** Clustering of meningiomas based on RNA-Seq data is presented using t-distributed stochastic neighbor embedding (t-SNE) of the first five eigenvectors obtained from spectral clustering. Each sample is represented by a number (for cluster membership) and color (for underlying driver event). A curve that encloses the samples belonging to the cluster 1 (named as Hh cluster) is drawn for illustration purposes. **b** A volcano plot of the differential expression (DE) tests for the Hh cluster against other meningiomas is shown. Red points show DE genes in the gene co-expression module M5 (*n* = 112). The top 20 genes ranked by *s*-values based on the local false sign rate (*s*_LFSR_) are labeled. Volcano plots for other clusters are presented in Supplementary Fig. 6. **c** Module eigengene distribution across transcriptional clusters for the module M5 is shown. Only the Hh cluster showed significant association with M5 (linear regression using 42 biologically independent samples, Benjamini-Hochberg corrected *P*-value = 1.2 × 10^−14^, Supplementary Fig. 7 and Supplementary Data 14). A boxplot indicates median (middle line), the first (Q1) and the third (Q3) quartiles (box), the smallest value down to Q1–1.5 IQR and the largest value up to Q3 + 1.5 IQR (whiskers), where IQR = Q3–Q1. Values beyond the end of the whiskers are plotted individually (outliers). Points are jittered horizontally to avoid overlaps. **d** Size factor-normalized read counts^76^ are plotted against meningioma driver subgroups for *IHH* and *SSH*. Expression of *IHH* and *SHH* were elevated among samples with SVs on 2q and 7q, respectively. On top of each panel, *s*_LFSR_ and posterior log2 fold change (postLFC) from DE analysis (Hh vs. others) are shown. TD, tandem duplication. CTX, inter-chromosomal translocation. CNV, copy number variation. CT, chromothripsis. Source data are provided as a Source Data file.

We next investigated how the SVs on 2q35 and 7q36.3 affect gene expression patterns of meningiomas using RNA-Seq, seeking to understand the molecular consequences of these events. We found that all the samples that acquired those SVs clustered with meningiomas harboring known Hh activating events (Fig. 3a, cluster 1, which we call the Hh cluster), such as a recurrent mutation *SMO*^*L412F*^ and a damaging one in *PTCH1* (ENST00000331920.6:c.1503+3A>G). This suggested that SVs on 2q35 and 7q36.3, as well as unidentified genomic or epigenomic alterations of five meningiomas that lacked established driver events, are associated with Hh signaling activation (Fig. 3a and Supplementary Fig. 5).

To investigate expression profiles of the Hh cluster (as well as other transcriptional clusters), we performed differential expression (DE) and gene co-expression network (GCN) analyses (Methods, Supplementary Figs. 6, 7, and Supplementary Data 12–17). Top DE genes for the Hh cluster (ranked by *s*-values based on the local false sign rate (LFSR)^33^) consistently belonged to a single network module M5 (80% of the top 30 DE genes that were also subjected to GCN analysis; Fig. 3b and Supplementary Data 17), which was most significantly associated with and was upregulated in this cluster among the 77 modules (Fig. 3c and Supplementary Data 14). 84.2% of the genes in M5 consisted of significantly DE genes for the Hh cluster, including canonical Hh pathway markers *HHIP*, *PTCH1*, and *GLI1* (ref. ^34^) (Supplementary Data 15 and Supplementary Fig. 8). Gene set overrepresentation analysis revealed that ‘Negative Regulation of Smoothened Signaling Pathway’ (GO:0045879) was the highest enriched biological process among the genes in this module (consistent with known feedback mechanisms associated with Hh signaling activation; Benjamini-Hochberg corrected *P*-value = 7.6 × 10^−4^; Supplementary Data 16). M5 did not exhibit a significant association with any of the other meningioma subgroups (Supplementary Data 14). Collectively, our RNA-Seq results provide strong evidence for activation of Hh signaling among meningiomas that harbor relevant genomic mutations in *SMO* or *PTCH1*, or events near 2q35 or 7q36.3.

We observed markedly elevated expression of *IHH* and *SHH* in the samples with SVs affecting 2q35 and 7q36.3, respectively (Fig. 3d). The meningioma that was identified to acquire a focal gain at 7q36.3 using WES (MN-60924, SHH-TD in Fig. 3d) also exhibited upregulation of *SHH*. It is notable that among the four genes (*FEV*, *CRYBA2*, *CFAP65* and *IHH*) where the copy numbers were preserved or gained (see above), only *IHH* showed a consistent upregulation of all the samples that acquired 2q35 events (Supplementary Fig. 9). In addition, genes telomeric to *IHH* up until the identified breakpoints also showed ectopic expression in tandem duplication cases (IHH-TD) and an inter-chromosomal translocation case (IHH-CTX, MN-52288, Supplementary Fig. 9). This suggested that the SVs could disrupt normal chromatin structure in this region, resulting in transcriptional dysregulation and potentially new enhancer-promoter contacts. In four of the five mutation-negative tumors that belonged to the Hh cluster, ectopic expression of *IHH* (*n* = 1) or *SHH* (*n* = 3) were observed (Fig. 3d). As this finding suggested that these meningiomas may also harbor SVs at 2q35 or 7q36.3, we performed low coverage tumor-only WGS for two of these samples (MN-61306 for the event near *IHH* and MN-61486 for that near *SHH*). This identified an inter-chromosomal translocation for the latter meningioma, with one of the breakpoints localizing to the region between *SHH* and *LMBR1* (Table 1). These results imply that events on 2q35 and 7q36.3 result in dysregulation and aberrant expression of Hh ligand molecules, leading to activation of the Hh signaling pathway.

**Fig. 4:**
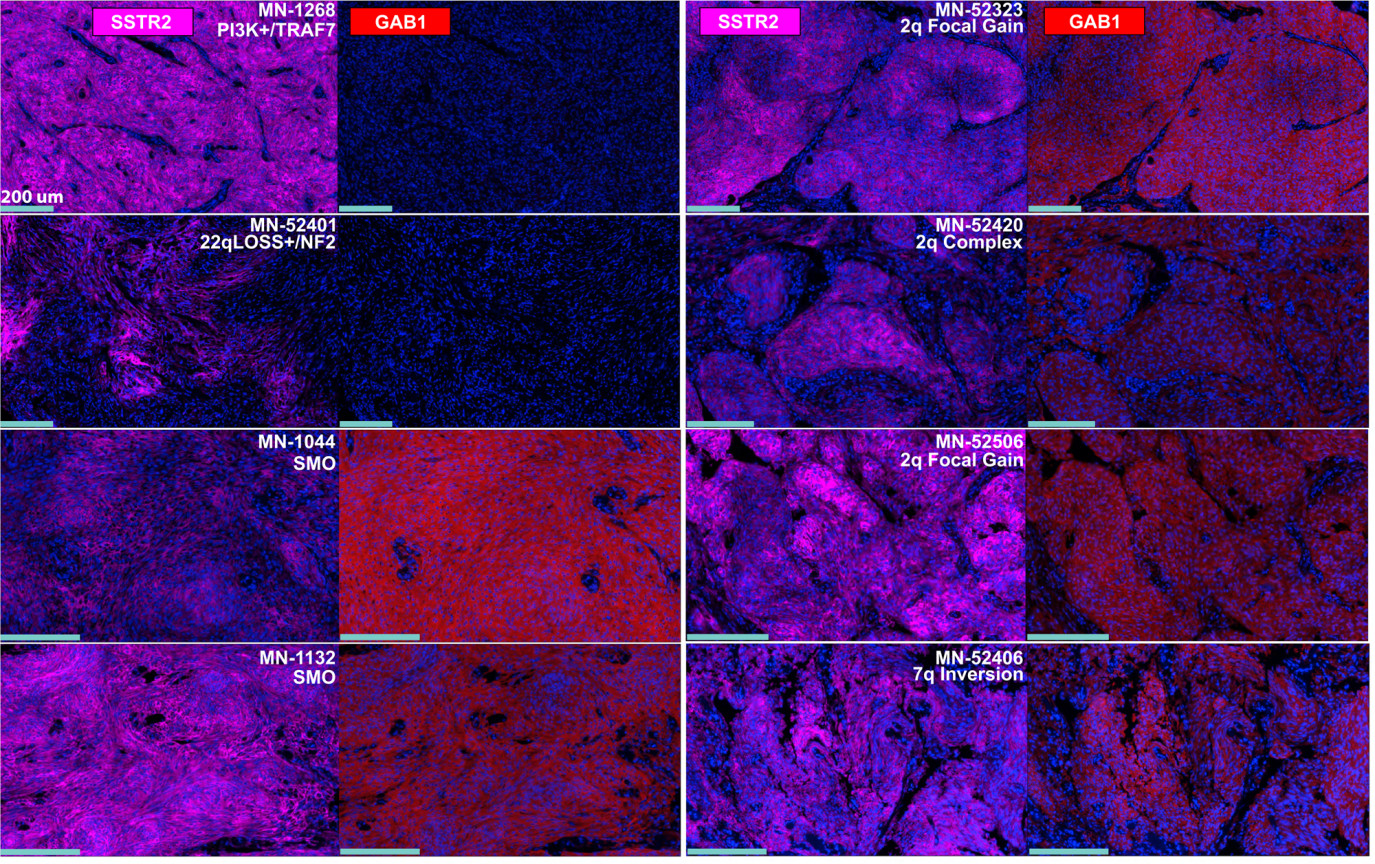
Hedgehog pathway activation in meningiomas with SVs on 2q35 and 7q36.3. Meningiomas with predicted Hh pathway activating events, including recurrent variants in *SMO* and SVs on 2q35 and 7q36.3, exhibit tumor-specific staining of GAB1^23,35^, a marker of Hh signaling activity. By contrast, we did not observe the staining of GAB1 in meningiomas with previously established non-Hh mutations. SSTR2 is a marker for meningioma cells^36^. The light blue scale bar in each image represents 200um. An additional 5 cases are shown in Supplementary Fig. 10.

To confirm our transcriptional results, we examined Hh activation patterns using multiplexed sequential immunofluorescence (seqIF™) of FFPE-preserved tumor tissues from various genomic subgroups (Fig. 4 and Supplementary Fig. 10). We found that meningiomas with predicted Hh signaling activating events (including recurrent *SMO* variants and structural events on 2q or 7q) exhibited robust staining of GAB1, a common clinical marker used to identify Hh pathway activation^23,35^. This staining was found specifically in tumor cells, and not in surrounding stroma, as evidenced by co-localization with SSTR2, a marker for meningiomas^36^. By contrast, no GAB1 staining was observed in meningiomas with non-Hh drivers. These results confirm that meningiomas with structural variations on 2q and 7q exhibit tumor-specific activation of the Hh signaling pathway at the protein level.

### Enhancer hijacking drives *IHH* expression in tandem duplication cases

To understand the molecular basis of the ectopic expression of *IHH* within the duplicated region, we next investigated the consequences of tandem duplications on chromatin regulation. Using HiChIP, we established three-dimensional contact maps, comparing two tandem duplication cases of *IHH* (TD set) and three non *IHH*-associated cases (control set). We identified significant chromatin interactions using FitHiChIP^37^, and topologically associated domains (TADs) using SpectralTAD^38^ (Methods). In addition, we performed H3K27ac ChIP-seq analysis for 37 samples to identify super-enhancers (Methods).

Notably, we identified a cluster of super-enhancers in an interval between 217 Mb and 219 Mb of chromosome 2, which was common among meningiomas (Supplementary Fig. 11). Two of these were found to exhibit tissue-specificity, being enriched in meningiomas but relatively absent in datasets reported for other tissues (Supplementary Fig. 12), and both overlapped a long non-coding RNA gene, *DIRC3*. Other super-enhancers in this interval overlapping with *IGFBP2* and *TNS1* were more ubiquitously expressed across different tissues. In the HiChIP control set, these super-enhancers interacted with regions within the same TAD and their neighbors (Supplementary Fig. 13). The region between 219 Mb and 220.25 Mb was relatively silent in terms of chromatin interactions, and a 50 kb interval that contained *IHH* (from 219.90 Mb to 219.95 Mb) did not show any significant contact with other intervals (at a 1% FDR, the minimum *q*-value = 0.0675 for this interval). We also did not observe any peaks as well as super-enhancers around *IHH* in any of the meningioma samples (Supplementary Fig. 11).

**Fig. 5:**
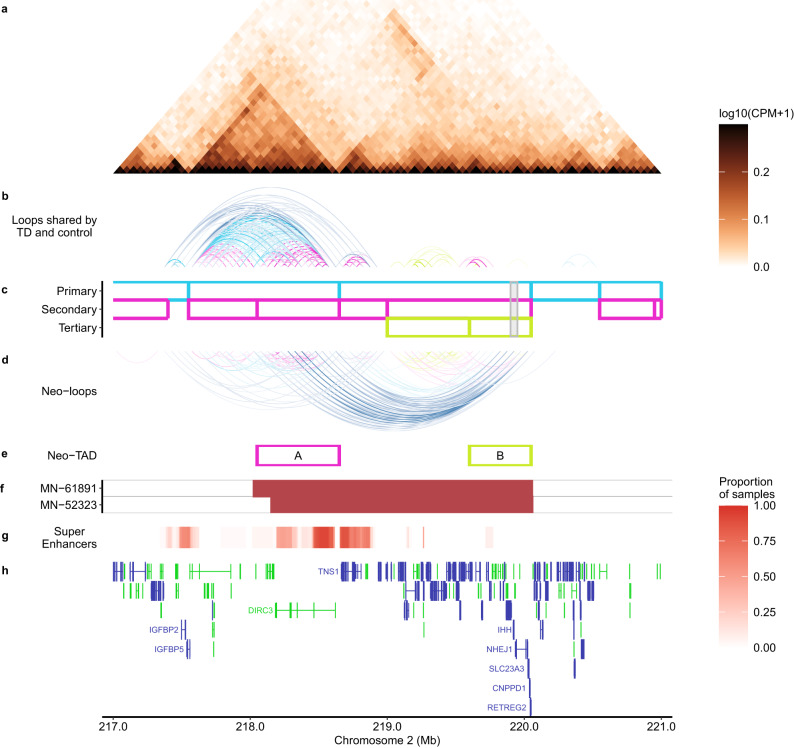
Tandem duplication involving *IHH* alters chromatin structure and creates neo-loops between *DIRC3* super-enhancers and *IHH*. HiChIP data of TD samples indicates the emergence of TAD boundaries that were not found in the control set (Supplementary Fig. 13), permitting interaction of *DIRC3* super-enhancers with the IHH locus. **a** HiChIP map, colors are based on the logarithm (base 10) of counts per million (CPM). **b** Significant loops shared between TD and control sets. **c** TAD boundaries detected by SpectralTAD. **d** Significant loops found only in the TD set (neo-loops). **e** TADs at edges of the tandem duplication segments (shown below). Their boundaries are not found in controls (Supplementary Fig. 13). It follows from the neo-loops that they form a neo-TAD such that domain B of the left copy and domain A of the right copy are juxtaposed ([A-B][A-B]) as a result of the TD. Another representation of this neo-TAD is presented in Supplementary Fig. 14. **f** Location of the tandem duplications for the samples included in the TD set. **g** Proportion of meningiomas in our H3K27ac ChIP-seq cohort (*n* = 37) that harbor an overlapping super-enhancer in the designated region. **h** Gencode basic genes (v38lift37) in this region. Genes overlapping the common super-enhancers as well as *IHH* are labeled. In addition, genes that exhibited ectopic expressions and were mapped upstream of *IHH* in tandem duplication cases are also labeled (Supplementary Fig. 9). Source data are provided as a Source Data file.

Compared with the control set, the TAD structure in the TD set was altered (Fig. 5) such that new TAD boundaries emerged and were dictated by the tandem duplication boundaries (Supplementary Fig. 14). Additionally, new significant loops emerged between *DIRC3* super-enhancer region (218 Mb-218.7 Mb) and a region around *IHH* (219.8 Mb-220.1 Mb), which were located adjacently because of the tandem duplication (Fig. 5, Supplementary Fig. 15, and Supplementary Data 18). Thus, a neo-TAD that includes both *DIRC3* super-enhancers and *IHH* was created (Supplementary Fig. 15), and the new enhancer-promoter contacts resulted in the ectopic expression of *IHH* as well as genes telomeric to it (Supplementary Fig. 9). A similar phenomenon was observed for the two cases of inter-chromosomal translocations (MN-52288 and MN-63401), whereby the chromosomal segments juxtaposed near *IHH* harbored super-enhancers near the breakpoints (Supplementary Figs. 16, 17). This mechanism is consistent with recent reports of enhancer hijacking, in which (super-) enhancers that are normally constrained within insulated TADs are juxtaposed near constitutively repressed oncogenes by SVs, resulting in aberrant activation of the oncogenes^39,40^.

## Discussion

Recent studies have used integrated approaches to classify meningiomas into clinically relevant subgroups^2,4,41^, leveraging multi-modal characterization including epigenomic, transcriptional, and genomic data. These reports built upon previous whole-exome and targeted sequencing projects that identified mutually exclusive genomic drivers in up to 80% of cases^11,12,14^, including previously unknown neoplasia genes such as *POLR2A*, *KLF4*, and *TRAF7*. However, despite a growing understanding of which patients harbor aggressive lesions, the genomic drivers of one-fifth of patients have remained obscure, preventing the use of precision medications with high efficacy.

In the present study, we performed a systematic analysis of SCNAs using a large cohort of WES and a SV analysis using WGS, which enabled us to identify mutually exclusive subgroups of low-grade meningiomas (Supplementary Fig. 18). Of the four mutually exclusive SCNA subgroups (Fig. 1), one was remarkable for multiple whole-chromosomal copy number gains (including majority of angiomatous and microcystic meningiomas), whereas the remaining three were characterized by losses of 22q, 3p or 2q. While 3p-loss has been reported specifically in higher-grade aggressive meningiomas^27,28^, this event comprised mostly WHO grade I (80%) meningiomas in our cohort, and often co-occurred with 1p-loss (88.2%). The fourth group consisted of complex 2q-losses that preserved a short genomic segment containing *IHH* (Fig. 2a, b). These three meningioma driver groups comprise mostly tumors originating from non-skull base locations (Supplementary Data 1). Among the remaining mutation-negative samples, we identified a cluster of focal gains at 2q35 encompassing *IHH* (*n* = 8) and four meningiomas that acquired SCNAs on 7q, including chromothripsis, complex gains and a focal gain at 7q36.3 involving *SHH* (Fig. 2c, d).

Further analysis of mutation-negative meningiomas using WGS identified copy-neutral SVs affecting 2q35 and 7q36.3 as well as specific event types behind the observed SCNAs (Table 1). Among the 13 tumors selected for WGS that were mutation-negative after WES analysis, four acquired SVs associated with *IHH* or *SHH*, and one acquired a damaging mutation in *DYRK1B*. We found three other tumors acquired SVs of unknown significance, including a tandem duplication involving *GLI1* (as a part of chromothripsis), *FOSB*-*LMNA* fusion and *EWSR1*-*CREB1* fusion. The second event was listed in a previous meningioma study^12^, while the third one in various neoplasms.

These results substantially increase the coverage of genomic alterations that may explain meningioma oncogenesis, at least for lower grade cases. Our WES cohort included 91 mutation-negative meningiomas (Supplementary Data 1). Among these, we found that 56 belong to previously uncharacterized SCNA subgroups (2q, 7q, 3p, and multiple whole-chromosomal gains) and one acquired *PTCH1* mutation (confirmed an association with Hh signaling activation, Fig. 3a and Supplementary Fig. 8), leaving 37% (34/91) to be mutation-negative. Therefore, together with our WGS result described above (9 remained unknown, including alterations with unknown significance), we estimate that, in this study, we were able to identify the candidate driver alterations in 74% (1 – 34/91 × 9/13) of the meningiomas that lacked somatic alterations in previously known drivers. In particular, the SVs associated with Hh ligands explained the molecular basis of 35.6% (22/91 + 34/91 × 4/13) of the previously mutation-negative meningiomas in our cohort. As with other genomic landscaping studies, future work is essential to validate the sufficiency of these alteration to drive meningiomagenesis.

Using RNA-Seq, we demonstrated that meningiomas acquiring SVs on 2q35 and 7q36.3 led to ectopic expression of the Hh ligands, *IHH* and *SHH*, respectively, and Hh pathway activation (Figs. 3 and 4). Transcriptional and multiplexed immunofluorescence results between Hh ligand and *SMO* mutant meningiomas were indistinguishable based on interrogation of well-established Hh signaling markers. We further characterized the meningiomas that acquired a tandem duplication involving *IHH* using H3K27ac ChIP-seq and HiChIP, discovering that the tandem duplication is associated with super-enhancer hijacking by *IHH* (Fig. 5). These observations expand the landscape of pharmacologically targetable low-grade meningiomas, while also implicating super-enhancer hijacking as a route to Hh-associated oncogenesis. In addition, we found mutations in Hh pathway genes including *PTCH1* and *DYRK1B*, which are promising candidate drivers. These results may thus provide a template for the study of other Hh-associated tumors that lack previously known genomic drivers.

The precise and potent role of Hh ligands in embryonic development necessitates careful epigenetic regulation in adult tissues, providing a fertile route for oncogenic disruption. Tandem duplications near the *IHH* locus have previously been implicated in ectopic expression of this gene in germline pathologies such as polysyndactyly and craniosynostosis due to the creation of novel enhancer-promoter contacts^18,19,42^. As in those disorders, the meningioma-specific events we have discovered exhibit remarkably similar boundaries, suggesting that precise breakpoints are necessary to enable de novo super-enhancer interactions with *IHH* (as has been suggested in other forms of cancer^43,44^). Notably, Hh pathway ligand overexpression has previously been observed in several forms of neoplasia^20,21^, however, the genomic events associated with this phenomenon have not been described in cancer. Given the large footprint of Hh signaling activity across development and neoplasia, it is possible that enhancer-associated ligand expression could prove to be the underlying molecular mechanism in other tumor types. Future genomic studies focusing on diverse Hh-associated cancers are needed to understand the full prevalence of this mechanism, particularly in samples lacking known alterations in this pathway.

Previous work by our lab and others identified robust associations of meningioma genomic drivers and tumor locations, whereby samples with bi-allelic loss of *NF2* tend to occur in the cerebral convexities and spine, while other meningiomas are enriched in the skull base^11,14,45^. Recurrent somatic mutations in *SMO*, which represent the primary route to Hh pathway activation in meningiomas, were observed in tumors originating from the midline anterior or middle cranial fossa^14^, an area where this pathway is embryologically active in development of the brain and other tissues^46,47^. Previous studies have hypothesized that the meninges in this region may harbor selective sensitivity to Hh pathway activation^48,49^, which may relate to a role in the development or proliferation of this tissue. Consequently, these cells may retain molecular features that confer sensitivity to Hh pathway activating mutations later in life, while meningeal cells of the cerebral convexities and other regions may be relatively unresponsive to molecular effects of *SMO* mutations acquired somatically. Similar anatomical explanations have been proposed for ameloblastomas and gliomas^50–52^.

Interestingly, we found that while *IHH*-activating meningiomas due to tandem duplications (*n* = 8, Supplementary Data 8) or simple translocations on 2q35 (*n* = 2, Table 1) were all found in the traditional skull base Hh niche, those due to complex rearrangements (chromothripsis or those cannot be described as a single SV type) were typically found in non-skull base or lateral middle cranial fossa (8 of 11, Supplementary Data 6; Fisher exact test *P*-value = 0.001). Regarding the *SHH*-activating meningiomas (*n* = 7), while one of the three complex rearrangements was found in a non-skull base meningioma, all simple SVs (*n* = 4) were found in skull base ones (*P* = 0.43; Table 1, Supplementary Data 1 and 10). The distinct genomic profiles of *IHH*-activating meningiomas in these locations suggest the existence of epigenetically unique cell populations, whereby differences in chromatin state confer variable sensitivities to specific genomic events. The complex rearrangements found in *IHH*-activating meningiomas result in marked chromosomal disruption and resulting changes in epigenetic regulation may induce Hh pathway sensitivity in an otherwise resistant cell population. Although we confirmed Hh signaling activation for two non-skull base meningiomas with chromothripsis on 2q (MN-52420 and MN-52336; see Fig. 4 and Supplementary Fig. 10 respectively), further study is required to gain mechanistic insights (such as whether it is attributable to enhancer hijacking) for this type of alterations. By contrast, meninges of the anterior and middle skull base, which appear to depend on Hh pathway activation during embryonic development, may be epigenetically primed to respond to this signaling pathway later in life, requiring merely a single tandem duplication or a translocation to overcome the suppression of ligand expression and pathway activation. Future experiments that investigate the epigenetic state of normal meninges in these regions are essential to understand spatial distribution of different types of SVs near *IHH* and may unlock important insights into the chromatin dynamics needed for Hh pathway responsiveness.

The role of Hh pathway activation in meningiomas was described more than a decade ago^11,12^, and subsequently spurred clinical-interest in the use of FDA-approved pathway inhibitors as treatment in surgically-refractory cases^53^. Understanding the importance of Hh pathway activation in the formation of meningiomas enables unique opportunities for single-agent chemotherapeutic agents, which may be particularly efficacious as meningiomas originate outside the blood-brain barrier (BBB). In most cases, Hh-activated meningiomas are histologically classified as WHO grade I, and do not exhibit the genomic or epigenetic changes recently found to be associated with aggressive behavior, such as loss of chromosome 1p or classification into aggressive methylation subgroups^2,4,5,54,55^. Nonetheless, Hh pathway-activated meningiomas occur in surgically challenging areas of the skull base and may recur in some patients^3^, emphasizing the need for improved non-surgical therapies. Previous clinical investigations have focused on *SMO*-mutated samples, which harbor recurrent L412F or W535L variants that exhibit known resistance to Vismodegib and other well-tolerated FDA-approved medications^56^. In our study, we identify overexpression of pathway ligands as a route to Hh signaling activation in meningiomas, a mechanism that occurs upstream of SMO and is thus predicted to respond to Vismodegib (Supplementary Fig. 19). Future evaluation with clinical trials should assess the responses in patients in this molecular subgroup and may validate an important tool in the treatment of surgically refractive cases.

## Methods

### Sample processing

Institutional Review Board approval and written patient informed consents were obtained at all participating sites in which specimens were obtained, including Yale University (the principal site of this investigation), Bahcesehir University, Marmara University, University of Bonn, Hopital Pitie-Salpetriere, University Hospital of Cologne, University of Pittsburgh and Acibadem Mehmet Ali Aydınlar University. This study is in compliance with all relevant ethical regulations. The reported analyses included a mixed cohort of newly characterized and previously reported meningiomas^3,11,14,45,57^. No patients were compensated for participation in this study. Our study population is a representative cross-section of mutation-negative meningiomas at several large academic medical centers. Sex and gender were not considered during study design because of the limited availability of samples. Surgical specimens were pathologically confirmed and were flash frozen for genomic study. When possible, a matching blood sample was obtained from each participating patient. Genomic material was extracted using the Allprep DNA/RNA Mini Kit (Qiagen), and its quality was assessed using a Bioanalyzer 2100 (Agilent). Demographic and clinical information of each subject are provided in Supplementary Data 1 based on the availability of medical records.

### Whole exome sequencing

#### Sequencing and preprocessing

Genomic DNA from reported samples underwent whole-exome sequencing (WES) as described previously and below^11,14^. The SeqCap EZ Exome v2.0 (Roche Life Science), xGen Exome Research Panel (Integrated DNA Technologies), or SeqCap EZ MedExome (Roche Life Science) kit was used for capture. After library preparation, sequencing was performed on the Illumina HiSeq platform (RRIDs: SCR_016386, SCR_016383, SCR_016387) with paired-end 74 or 100 base pair reads. The tumor and blood samples were sequenced to a target depth of 185 and 85 reads, respectively. Sequenced reads were aligned to the human reference genome (GRCh37) using BWA-mem (version 0.7.15)^58^. PCR duplicates were marked with Picard (version 2.17.11, RRID:SCR_006525)^59^, followed by local realignment and base quality recalibration using Genome Analysis Toolkit (GATK, version 3.4, RRID:SCR_001876)^60^.

#### Somatic copy number alteration (SCNA) calling

Depths of coverage of target intervals for each sample were calculated by using GATK and they were converted to coverage ratios between tumor and blood samples. Alternate allele frequencies (or B allele frequencies, BAFs) of tumor and blood samples were estimated at biallelic single nucleotide variant (SNV) sites for blood samples using SAMtools (RRID:SCR_002105)^61^ and retained the sites with the coverage of blood sample ≥ 8 and blood BAF being between 1/3 and 2/3. We used the variation of the deviation of BAF from its mean (devBAF) within a segment to evaluate the loss of heterozygosity (LOH) or allelic imbalance. Copy number states were inferred by the ExomeCNV R package^62^ (RRID:SCR_010815) using the logarithm (base 2) of the coverage ratios (logCR). We excluded a segment if more than 70% of the bases overlapped low-complexity region(s), downloaded from https://github.com/lh3/varcmp/blob/master/scripts (ref. ^63^).

#### Merging consecutive segments

As the segmentation of ExomeCNV was aggressive and sensitive to occasional noise in the coverage ratio, we sequentially concatenated two consecutive segments when the event types (deletion, amplification, or neutral) were the same, logCR was within ± 0.05 each other, and the mean of devBAF was within +/- half of the standard deviation of devBAF each other. In addition, if either or both segments contained less than three SNV sites and were inferred to have the same event type, they were always merged.

#### Final SCNA call

We tested for the LOH or allelic imbalance of each merged segment using two methods, the Ansari-Bradley test (one-sided) to evaluate if the variance (scale) of tumor devBAF is greater than that of blood, and a Z-score test for tumor sample (Z = (mean devBAF) / (standard deviation of devBAF), two-sided) to evaluate the non-random directional shift of devBAF from zero. The final copy number state for each segment was determined as follows (*P*_ABT_, *P*-value of the Ansari-Bradley test; *P*_dev_, *P*-value of the Z-score test for tumor devBAF; *M*_dev_, the mean devBAF of the tumor sample; and n, the number of sites within the segment; *n*_T_, the number of target intervals within the segment; and logCR, log_2_ coverage ratio). When the ExomeCNV call was deletion, we classified the segment to LOSS if (*P*_ABT_ < 0.05 & *P*_dev_ < 0.1 & *M*_dev_ ≥ 0.1) or (*P*_ABT_ ≥ 0.05 & *P*_dev_ < 0.05 & *M*_dev_ ≥ 0.15) or (*n* > 0 & *n* < 3 & *n*_*T*_ ≥ 10 & *M*_dev_ ≥ 0.17). When the ExomeCNV call was amplification, we classified the segment to GAIN if (*P*_*ABT*_ < 0.01 & *n* ≥ 20 & logCR > 1.11) or (*P*_*ABT*_ < 0.05 & *P*_*dev*_ < 0.1) or (*P*_ABT_ ≥ 0.05 & *P*_dev_ < 0.05 & *M*_dev_ ≥ 0.1) or (*n*_T_ ≥ 100 & CR ≥ 1.3). For focal gains at 2q35 (see main text), we also looked for amplification events with *n*_T_ ≥ 60 and *M*_dev_ ≥ 0.08 and called them ‘GAIN-filtered’ (Supplementary Data 7). Finally, when the ExomeCNV call was neutral, we classified the segment to copy-neutral LOH (CN-LOH) if (*P*_ABT_ < 0.05 & *P*_dev_ < 0.1 & *M*_dev_ ≥ 0.15) or (*P*_ABT_ ≥ 0.05 & *P*_dev_ < 0.05 & *M*_dev_ ≥ 0.2).

#### Sample filtering

We considered that the presence of extremely fragmented segments and largely variable (or scattered) coverage-ratio estimates were indicative of large false positive and negative SCNA calls. We used the following conditions to identify potentially problematic samples: (1) the median absolute deviation of logCR > 0.2; or (2) the proportion of the segments that included less than 10 targets was >20%, and the median absolute deviation of logCR > 0.15 or the number of segments >3,000. Only those samples that passed the above filters were used in the statistical analysis (‘exome-pass’ in Supplementary Data 1, *n* = 251). After reviewing the failed samples (*n* = 42) by plotting logCR, tumor BAF and SCNA calls across genome, about half of them were benign such that we were able to reliably call some targeted SCNAs such as large 3p-loss or 22q-loss (‘exome-usable’, *n* = 22). The genomic driver status of such samples was used to label them in RNA-Seq and H3K27ac ChIP-seq data analysis. The remaining samples (‘exome-mutation’, *n* = 20) were used only to call mutations.

#### Size of SCNAs and categorization

Due to the noises in the coverage ratios, the segmentation performed by ExomeCNV often resulted in the division of a single event into multiple pieces even after the merging process described above. Therefore, we decided to estimate the size (length) of SCNAs by a total length of the same event type on the same chromosome arm. We called a large SCNA if the total length was longer than 9 Mb (Supplementary Fig. 1). We then categorized SCNAs by the event types (LOSS, CN-LOH, and GAIN) and the chromosome arms. However, to avoid duplicated counts for whole-chromosomal events such as aneuploidy, we added categories without arm designations and classified events that occurred and covered at least 50% of both arms into these new categories.

#### Mutual exclusivity and co-occurrence tests

To identify driver SCNAs, we performed mutual exclusivity and co-occurrence analysis for large SCNAs using Discrete Independence Statistic Controlling for Observations with Varying Event Rates (DISCOVER)^64^. Statistically significant mutually exclusive or co-occurring event pairs were selected at a 5% false discovery rate (FDR). Some categories were merged to increase efficiency and avoid duplicated counts. We merged whole chromosomal losses ‘1-LOSS’, ‘3-LOSS’, ‘7-LOSS’, ‘4-LOSS’, ‘6-LOSS’ and ’18-LOSS’ into ‘1p-LOSS’, ‘3p-LOSS’, ‘7p-LOSS’, ‘4p-LOSS’, ‘6q-LOSS’ and ‘18q-LOSS’, respectively. Whole-chromosomal CN-LOHs were counted together with whole-chromosomal gains, which might be a reasonable assumption because recovering the copy-neutral state (that requires chromosomal gain by various mechanisms) was more favored than maintaining the state of chromosomal loss that is presumed to occur initially. In addition, as previous studies demonstrated that the biallelic loss of *NF2* is the driver alteration, we excluded 22q loss that did not overlap *NF2*.

#### Somatic mutation calling and filtering

Somatic variants, including single nucleotide variations (SNVs) and small insertion/deletions, were called using Mutect2 implemented in GATK3.5. Mutations were filtered using Mutect2 default values. Further, we excluded those that showed either (i) the read orientation bias in the tumor sample (defined by the proportion of F1R2 reads for indels or SNVs with the reference base G or T, or of F2R1 reads for SNVs with the reference base A or C being less than 0.05 or greater than 0.95); (ii) the low base quality of alternate allele in the tumor sample (<25 in the Phred scale); (iii) the low tumor variant allele frequency calculated directly from allelic depths annotations (which is different from estimated allele frequency of Mutect2) < 0.02; (iv) the low base-quality-weighted alternate-allele frequency ratio between tumor and normal samples when there was at least one read supporting alternate allele in the normal sample, (AF_ALT_tumor × BQ_ALT_tumor) / (AF_ALT_normal × BQ_ALT_normal) <4, where AF_ALT stands for the alternate allele frequency estimated from allelic depth and BQ_ALT the alternate allele base quality; or (v) the low reference base quality in either tumor or normal sample (<20 in the Phred scale). The resulting mutations were annotated using Variant Effect Predictor (v99)^65^ and CADD (v1.4, RRID:SCR_018393)^66^, including population frequency in control databases (gnomAD, RRID:SCR_014964)^67^. Samples were considered to be mutation-negative if they lacked previously reported somatic variants in *NF2* (or chromosome 22q loss), *TRAF7*, *KLF4* (K409Q), *AKT1* (E17K), *PIK3CA*, *PIK3R1*, *AKT3* (E17K), *SMO* (L412F/W535L), *SUFU*, *PRKAR1A* (A17D), *POLR2A* (Q403K/L438_H439del), *SMARCB1*, or *SMARCE1*^11,12,14,28,68,69^.

#### Candidate driver mutations

We selected candidate driver mutations as follows: (i) protein-altering mutations according to ENSEMBL consequences (splice acceptor/donor variant, stop gained, frameshift variant, stop/start lost, inframe insertion/deletion, missense variant, protein altering variant, or splice region variant, see https://m.ensembl.org/info/genome/variation/prediction/predicted_data.html); (ii) mutations with CADD PHRED score > 14.1; (iii) tumor variant allele frequency ≥ 0.1; and (iv) mutations that are unlikely to be germline: the number of gnomAD populations that identified the mutation is less than two, and the allele frequency is not greater than 0.0001 at 95% confidence bound in any of the populations estimated from gnomAD exome sample sizes assuming Poisson distribution^70^.

### Whole genome sequencing

#### Sequencing and preprocessing

Meningiomas that lacked known drivers, as well as candidate driver SCNAs identified in this paper, or some of those that acquired SCNAs at 2q35 or 7q36.3 in the WES analysis, were selected for whole-genome sequencing (WGS). Genomic DNAs were obtained for 18 tumor-blood pairs and six tumor-only samples. Samples were selected for WGS that either: (1) acquired SCNAs at 2q35 or 7q36.3 based on WES (*n* = 9), (2) were mutation-negative and lacked candidate drivers after WES analysis (*n* = 13), or (3) exhibited ectopic expression of *SHH* or *IHH* (*n* = 2). WGS libraries were prepared using the Illumina TruSeq DNA PCR-Free sample preparation kit (Illumina, FC-121–3001) according to the manufacturer’s protocol. Data were acquired on the NovoSeq platform (Illumina), using paired-end, 150 base pair read. The target coverage depth for tumor and blood samples was 60x and 30x, respectively. Five of the tumor-only samples were targeted for 20x as they acquired SCNAs at 2q35 or clustered to the Hedgehog sample cluster in RNA-Seq data. Reads were processed using the same pipeline as WES.

#### Structural variant calling and filtering

To detect somatic structural variations, we used MANTA^71^. For paired samples, MANTA was run on tumor-normal mode. For tumor-only samples, we paired a tumor sample with all available blood samples, ran MANTA in the tumor-normal mode, and extracted variants called consistently in all the batches. We filtered detected variants by requiring that they showed the somatic (quality) score ≥ 50, they were not annotated as IMPRECISE, and there was no overlapping breakend in any of the blood samples (for paired samples). For tumor-only samples, as we still expected a high false-positive rate, we only considered the events within 2q35 and 7q36.3.

### RNA sequencing

#### Sequencing and preprocessing

RNA-sequencing (RNA-Seq) analysis was performed on a cohort of 42 meningiomas (19 previously reported^14^). The RNA Integrity Number (RIN) was determined using a Bioanalyzer 2100 (Agilent; RRID:SCR_019389) to ensure sufficient quality prior to sequencing. Each specimen underwent ribozero depletion and was sequenced on the Illumina HiSeq platform (RRIDs: SCR_016386, SCR_016383, SCR_016387) using paired-end, 75 base pair read. Adaptor contamination was removed from all reads using cutadapt (v0.9.4, RRID:SCR_011841)^72^. Additional trimming was performed on low-quality 3’ bases and reads with a final length of less than 35 base pairs were removed. Data were aligned using STAR (v2.4, RRID:SCR_004463)^73^. Transcripts were quantified using Kallisto (RRID:SCR_016582)^74^ based on the GENCODE v34 transcriptome and with 100 bootstrap samples.

#### Sample clustering

We summarized read count data per transcripts to genes using tximport R package^75^ and then normalized read counts using regularized logarithm transformation (rlog) implemented in DESeq2^76^. We then performed multidimensional scaling (MDS) and identified that the first dimension was highly correlated with experimental bias, namely, the logarithm of the ratio of intronic and target bases (Pearson correlation, *r* = −0.98), as well as the standard deviation of normalized expression values (*r* = 0.975), and the mean normalized expression values (*r* = −0.923). Therefore, we removed this unwanted variation by regressing the normalized expression values onto the MDS first dimension for each gene. We used the residuals obtained as such to cluster samples. We then selected, from 60,669 genes, 14,687 (24.2%) autosomal genes that were not annotated to be rRNA or rRNA pseudogene and showed large variability (number of samples with zero expression <2, log_2_(2) < (the maximum normalized count) <(the maximum of the log_2_ raw count) + 2, and the standard deviation of residuals >0.38). We obtained a sample similarity (adjacency) matrix by calculating inner products of residual expression vectors (Gram matrix) after mean centering followed by scaling the matrix such that the largest absolute of the entire matrix was unity. We applied a spectral clustering method (Ng-Jordan-Weiss algorithm^77^) to the similarity matrix and determined the number of clusters (5) by using the eigengap method. To determine cluster memberships, we applied Gaussian mixture model clustering (ClusterR package, https://CRAN.R-project.org/package=ClusterR) to the top five eigenvectors. To visualize the clustering result in the 2-dimensional plane, we applied the t-distributed stochastic neighbor embedding (t-SNE) to the top five eigenvectors.

#### Differential expression analysis

We defined seven transcriptional clusters by splitting the clusters 2 (2_PI3K and 2_KLF4) and 4 (4_22qLOSS and 4_3pLOSS) into two subclusters based on the driver alterations and eigenvectors, respectively (Supplementary Fig. 5, Supplementary Data 1 and Fig. 1). We performed differential expression analysis using DESeq2 (ref. ^76^) framework. We compared each cluster with others. We retained genes that were detected in at least one sample and excluded potential duplicates (homologous genes) based on the count data. We tested differential expression using the maximum likelihood estimates (Wald test, the results function of DESeq2 with default setting). After excluding genes with the standard error of log2 fold change (LFC) being zero and those with low count genes identified by independent filtering, we performed empirical Bayes shrinkage estimation of LFC and testing using lfcShrink function with apeglm method^78^ that was modified to use replaced counts when there were outliers (so that the results of lfcShrink and the results function become consistent). We obtained two *s*-values based on the local false sign rate (*s*_LFSR_)^33^ and the local false sign or smaller (*s*_LFSOS_) rate^78^. The former *s*-values were used to rank genes while the latter were used to evaluate the significance. To estimate the *s*_LFSOS_, we set the LFC threshold at log_2_(1.2) such that genes with very small effect size would not be called significant. The significance level of *s*_LFSOS_ was set to be 0.01.

#### Gene co-expression network analysis

To construct gene co-expression network (GCN), we selected 10,000 most variable genes that had records in both ENSEMBL and Entrez. We obtained an adjacency matrix by calculating a robust correlation matrix using bicor function (biweight midcorrelation)^79^ with maxPOutliers = 0.05, setting negative correlation values to 0 (so-called “signed hybrid” method) and then normalizing the matrix^77^. We adopted the normalized lmQCM (local maximal Quasi-Clique Merger) framework^80^ to construct GCN. In contrast to WGCNA framework^81^, this method allows for a gene to belong to multiple network modules.

(1: module detection) We identified network modules using localMaximumQCM function implemented in lmQCM R package^80^ (steps 1–12 of the algorithm described in ref. ^80^, see below for a correction) with default parameters for *λ* = 1 and *t* = 1, while *γ* = 0.3 was determined by examining a curve of the number of modules against *γ* values as in Fig. 1 of ref. ^80^. We discard modules with size <4 after this step.

(2: module reduction) Identified modules were examined for overlaps and modules with the highest overlap were merged as described below (step 13 of ref. ^80^).

(3: module characterization) We calculated the module eigengene for each module that is the first principal component of the gene-wise standardized expression matrix of the genes in the module^81^. The module eigengene is an *n*-vector, where *n* is the number of samples, that describes the summarized expression level of all the genes in the module. Module eigengenes were aligned such that they are positively correlated with average expression. To evaluate the connectedness between genes within a module, we calculated the module membership (*k*_ME_) and intramodular connectivity (*k*_IM_)^81^.

(4: module pruning) We refined each module by removing genes with poor connectivity to other members based on lower module membership (*k*_ME_ < 0.5) or lower intramodular connectivity (scaled *k*_IM_ < 0.6), as well as those that showed negative correlation to any other gene in the same module. We then recalculated module variables following the step 3 above.

(5: module merging) We merged highly correlated modules to remove redundancy by calculating correlations between module eigengenes using bicor. We sequentially merged the most correlated module pair and recalculate module eigengenes at each iteration. This step was repeated until there is no module pair with bicor ≥ 0.8. Then we perform the module pruning as described above. We excluded modules with size <8.

(6: module expansion) For genes that were not members of any modules, we tested if any of those genes can belong to the identified modules by using the same algorithm as localMaximumQCM. We then followed steps 3, 4 and 5 above. We excluded modules with size <10.

A total of 77 modules of size 10 or greater were identified. For each of the seven meningioma subgroups, we tested for association with 77 module eigengenes using linear regression and found that 51 modules are associated with at least one cluster after multiple testing correction using Holm’s method^82^ at the significance level of 0.05. We also performed the gene set overrepresentation analysis using clusterProfiler^83^ and MSigDB (msigdb_v2022.1.Hs) for each module. We considered module members were significantly enriched for a given ontology/pathway if Benjamini-Hochberg corrected *P*-value < 0.05.

#### Modifications to lmQCM implementation

We modified the original implementation in the localMaximumQCM function implemented in lmQCM R package^80^, which discards a local maximal edge if both nodes have been members of any of the previously identified modules even when two nodes belong to different modules, to make an edge being discarded only when both nodes belong to the same module (correctly reflecting the step 3 of ref. ^80^). In addition, we implemented an alternative algorithm for the overlap-based module merging step (step 13 of ref. ^80^, implemented in merging_lmQCM function of lmQCM package) to maintain connectivity within a module by avoiding merging less similar modules as follows: (1) remove modules that are complete subset of any of the larger module; (2) sort modules by ascending order of sizes and then by the descending order of the maximum overlap, K(x) = max_y ∈ M_ (|intersect(x, y)|/|x|) for a module x, where M is a set of all modules, intersect(x,y) denotes the intersection of two modules and |.| stands for the number of elements (genes). We then have an ordered set of modules [x_i_: K(x_i_) ≥ K(x_i+1_)]; (3) merge the first module x_1_ to [x_i_: |intersect(x_1_, x_i_)|/|x_1_ | = K(x_1_), i > 1] if K(x_1_) ≥ β (0 < β < 1); and (4) iterate the above steps 2 and 3 until there is no module with K(x) ≥ β. We set β = 0.6.

### Automated multiplexed sequential Immunofluorescence (seqIF™) imaging

Automated hyperplex IF staining and imaging was performed on FFPE sections using the COMET™ platform (Lunaphore Technologies). The multiplex panel included antibodies for *SSRTR2* (Ab134152; 1/2000; Rabbit), a meningioma marker, and *GAB1* (Cell signaling Polyclonal 3232; 1/100; Rabbit), a marker for Hh pathway activation. The protocol was generated using the COMET™ Control Software, and reagents were loaded onto the COMET™ device to perform the seqIF™ protocol. All antibodies were validated using conventional IHC and/or IF staining in conjunction with corresponding fluorophores and 4’,6-diamidino-2-pheynlindole counterstain (DAPI, ThermoFisher Scientific). For optimal concentration and best signal-to-noise ratio, all antibodies were tested at 3 different dilutions, starting with the manufacturer-recommended dilution (MRD), MRD/2, and MRD/4. Secondary Alexa fluorophore 555 (ThermoFisher Scientific) and Alexa fluorophore 647 (ThermoFisher Scientific) were used at 1/200 and 1/400 dilutions, respectively. The optimizations and full runs of the multiplexed panel were executed using the seqIF™ technology integrated in the Lunaphore® COMET™ platform (characterization 2 and 3 protocols, and seqIF™ protocols, respectively). The seqIF™ workflow was performed parallelized on a maximum of 4 simultaneous slides, with automated iterative cycles of 2 primary antibodies’ staining at a time, followed by imaging, and elution of the primary and secondary antibodies. No sample manipulation is required during the entire workflow. All reagents were diluted in Multistaining Buffer (BU06, Lunaphore Technologies). Elution step, using Elution Buffer (BU07-L, Lunaphore Technologies), lasted 2 min for each cycle and was performed at 37 °C. Quenching step lasted for 30 sec and was performed with Quenching Buffer (BU08-L, Lunaphore Technologies). Imaging step was performed with Imaging Buffer (BU09, Lunaphore Technologies) with exposure times set at 4 min for all primary antibodies and secondary antibodies at 2 min. Imaging step is performed with an integrated epifluorescent microscope at 20x magnification. Image registration was performed immediately after concluding the IF staining and imaging procedures by COMET™ Control Software. Each seqIF™ protocol resulted in a multi-stack OME-TIFF file where the imaging outputs from each cycle are stitched and aligned. COMET™ OME-TIFF file contains DAPI image, intrinsic tissue autofluorescence in TRITC and Cy5 channels, and a single fluorescent layer per marker. Markers were subsequently pseudocolored for visualization of multiplexed antibodies using the Viewer software (Lunaphore Technologies).

### H3K27ac ChIP-sequencing

#### ChIP-seq experiment and preprocessing

Chromatin Immunoprecipitation followed by Sequencing (ChIP-seq) was performed as previously described^57^, with minor modifications. Frozen tumor specimens were sectioned into 15 µM slices and immediately cross-linked with 1% formaldehyde for 15 min. Samples were quenched with glycine for 10 min, then washed twice with chilled Phosphate Buffered Saline (PBS). Tissues were disrupted using dounce homogenization, then re-suspended in nuclear lysis buffer with 0.2% Sodium Dodecyl Sulfate (SDS). Samples were then sheared to achieve fragments of 100–300 base pairs using a QSonica Q800R sonicator (amplitude 70%). Chromatin was incubated overnight with beads coated in H3K27ac antibody (Abcam ab4729, RRID:AB_2118291), while 10% of the sample was saved as input for later sequencing. The next day, beads were washed with low and high salt buffers, then chromatin was eluted, and crosslinks were reversed. Samples were purified using the Qiagen PCR purification kit. Sequencing was performed on the Illumina HiSeq platform using single-end 75 base pair reads. Replicates for each genomic subgroup were included when available, and each replicate represented a distinct patient tumor specimen. ChIP-seq reads were trimmed to remove adaptor contamination and low-quality bases. The resulting reads were aligned using Bowtie 2 (RRID:SCR_005476)^84^. Among the samples reported in this paper (*n* = 37), 20 have previously been reported^57^.

#### Peak calling and super-enhancer detection

ChIP enriched peaks (narrow peaks) were identified using MACS2^85^. Super-enhancers were detected using Rank Ordering of Super-Enhancers (ROSE, RRID:SCR_017390)^86,87^ with a slight modification for efficiency: (1) Peak stitching step was re-implemented in R following the same algorithm of ROSE^87^; (2) Coverage calculation was performed using multiBamSummary implemented in deepTools (RRID:SCR_016366)^88^; (3) The total number of mapped reads was calculated using SAMtools flagstat for treatment and input samples, respectively; and (4) Super-enhancer detection was performed using R functions calculate_cutoff and numPts_below_line implemented in ROSE (R-script ROSE_callSuper.R).

### Hi-C followed by H3K27ac ChIP-seq (HiChIP)

#### HiChIP experiment

HiChIP was performed as previously reported^89^, with adjustments for use in frozen tumor specimens. Briefly, 15 to 30 slices (20 µm) of each flash-frozen tumor specimen were sectioned using a cryostat and collected in chilled PBS. Samples underwent dounce homogenization, then fixation in 1% formaldehyde for 15 min. The reaction was quenched in glycine for 10 min, then washed twice with chilled PBS. Pelleted samples were flash frozen in liquid nitrogen and stored for later use. For HiChIP, pellets were thawed on ice and incubated in Hi-C lysis buffer for 30 min with rotation. Nuclei were pelleted and washed again with Hi-C lysis buffer, then incubated in 0.5% SDS for 10 min at 62 degrees. The reaction was quenched in Triton X-100 for 15 min at 37 degrees. Samples were then incubated with MboI restriction enzyme (NEB, R0147) for two hours. MboI was heat-inactivated for 20 min (62 degrees), followed by extension of overhands using biotin-ATP (Thermo Fisher, 19524016) for 1 h. Fragments were ligated using T4 DNA ligase (NEB, M0202) for 4 h. Samples were then pelleted and re-suspended in Nuclear Lysis Buffer. ChIP-seq was then performed as described above, using an amplitude of 70% and duration of 10 min. After sonication, samples were diluted to reduce SDS concentration, then incubated with H3K27ac antibody (Abcam ab4729, RRID:AB_2118291) overnight (5 µg per sample). The next morning, 50 µL of washed beads were added to each tube and incubated for 2 h with rotation (4 degrees). Samples were washed with low-salt, high-salt, and LiCl buffer 3 times each at room temperature on the magnet. DNA was eluted then purified with DNA Clean and Concentrator columns (Zymo Research). Streptavidin C1 beads (Thermo Fisher) were washed with Tween Wash Buffer and resuspended in 2x Biotin Binding Buffer, then 5 µL of beads was added to each tube. Samples were incubated for 15 min shaking, then washed twice with Tween Wash Buffer. They were incubated at 55 degrees with shaking during each wash. Subsequently, samples were washed with 1x Tagment DNA (TD) buffer (Illumina), then re-suspended for the Tn5 transposase reaction. A total of 0.2 µL Tn5 was used for each sample, and they were incubated at 55 degrees with shaking for 10 min. Samples were then incubated with 50 mM EDTA for 30 min at 50 degrees, then two times each with 50 mM EDTA and Tween Wash Buffer. Samples were then washed with 10 mM Tris and underwent 10 rounds of PCR amplification using Nextera primers. After PCR, samples were placed on the magnet and the supernatant was collected and purified (Zymo Research). DNA was quantified using picogreen and balanced between samples prior to pooling. Two-sided size selection was performed using Ampure XP beads and paired-end sequencing was performed with 75 base pair reads on the Illumina platform. Greater than 70 million reads were obtained per sample. A detailed protocol including buffer recipes is reported previously^89^.

#### Hi-ChIP data analysis

Paired-end sequencing data were processed using the Hi-C Pro pipeline^90^, including alignment to hg19 using Bowtie 2 (ref. ^84^). For meningiomas without *IHH* (Indian Hedgehog) tandem duplications, data from three unique patients (MN-52407, MN-52430, and MN-60826) were combined, generating a total of 423.4 million paired reads. For meningiomas with *IHH* tandem duplications, data from 2 patients (MN-52323 and MN-61891) were combined, generating a total of 785.1 million paired reads. Fragments were mapped to MboI restriction sites using a digested reference genome generated by the ‘digest_genome.py’ script of Hi-C Pro. Iterative correction and eigenvector decomposition (ICE) normalization were performed on all datasets with a bin size (or resolution) of 50 kb. Topologically associated domains were inferred by using SpectralTAD^38^. Chromatin interaction loops were identified using FitHiChIP^37^ at a 1% false discovery rate (FDR). We used H3K27ac ChIP-seq peak calls from MN-52323 and MN-52407 for IHH tandem duplication and non-IHH data, respectively, as input for FitHiChIP. We adopted a “peak to all” option so that only pairs of bins in which at least one bin overlapped H3K27ac peak. To visualize the interaction matrix (Fig. 5a, Supplementary Figs. 13a and 15), we used GENOVA R package^91^.

### Sanger sequencing

Ambiguous WES variants were confirmed using Sanger sequencing. Primers flanking the mutations of interest were designed, and regions amplified via PCR on an ABI 9800 Fast Thermocycler. Primers sequences are available upon request.

### Reporting summary

Further information on research design is available in the Nature Portfolio Reporting Summary linked to this article.

### Supplementary information

Supplementary Information

Description of Additional Supplementary Files

Supplementary Data 1

Supplementary Data 2

Supplementary Data 3

Supplementary Data 4

Supplementary Data 5

Supplementary Data 6

Supplementary Data 7

Supplementary Data 8

Supplementary Data 9

Supplementary Data 10

Supplementary Data 11

Supplementary Data 12

Supplementary Data 13

Supplementary Data 14

Supplementary Data 15

Supplementary Data 16

Supplementary Data 17

Supplementary Data 18

Reporting Summary

### Source data

Source Data

## Data Availability

The publicly available RNA-seq and ChIP-seq data used in this study are available in the Gene Expression Omnibus database under accession code GSE85135^14^. The de-identified sequencing datasets generated as a part of this study, including RNA sequencing, whole-exome sequencing (new drivers), H3K27ac ChIP-seq and H3K27ac Hi-ChIP, are deposited in the European Nucleotide Archive, under accession code PRJEB55424. The remaining data are available within the Article, Supplementary Information, Supplementary Data or Source Data file. Source data are provided with this paper.
