# Supplementary material for: Super-enhancer hijacking drives ectopic expression of hedgehog pathway ligands in meningiomas
Source: Nat Commun. 2023 Oct 7;14:6279. doi: 10.1038/s41467-023-41926-y (PMC10560290; doi:10.1038/s41467-023-41926-y)
Supplement: Supplementary file 1 — Supplementary Information [file 41467_2023_41926_MOESM1_ESM.pdf]

SUPPLEMENTAL FIGURES

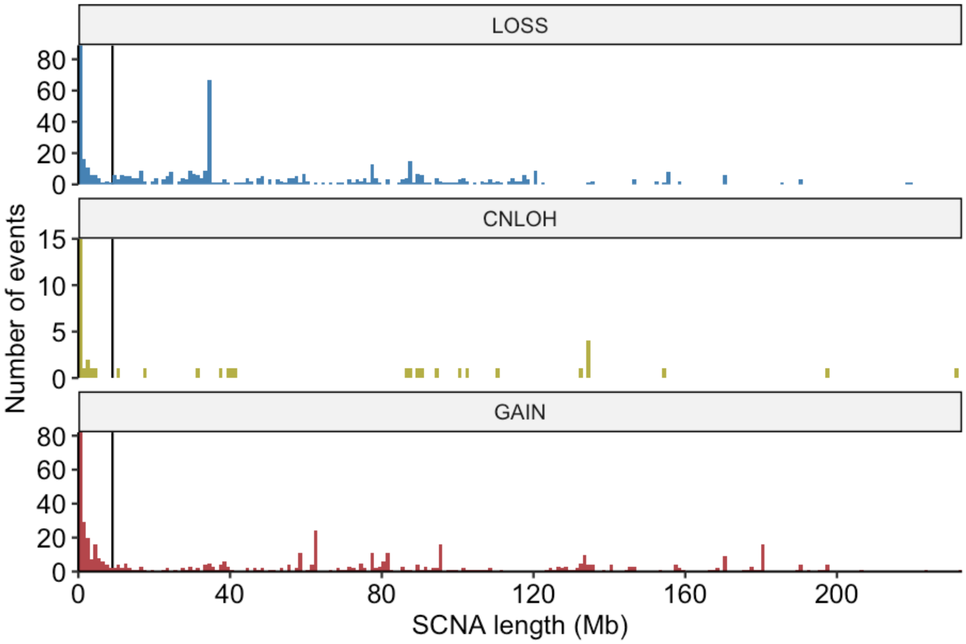

Supplementary Fig. 1: SCNA length distribution. Relationship between the SCNA lengths and their frequencies (per 1 Mb bins) for all the SCNAs observed in our cohort is shown. See Methods for the definition of the SCNA lengths. We defined a large SCNA as collected segments of the same event type (loss, CN-LOH and gain) on the same chromosome arm with a total length > 9 Mb (the cutoff is indicated by a black vertical line) by observing the inverse relationship between the length and frequency in the < 9 Mb range while there is no such relationship in > 9Mb. When both arms were covered > 50% by the same event type, these events were counted to be a single event on a chromosome so that the length for such an event is the sum of the covered lengths in p and q arms. The vertical axis of each plot shows the frequencies of each type of SCNAs across our cohort fell within the corresponding 1 Mb bins. SCNA, somatic copy number alteration; CN-LOH, copy-neutral loss of heterozygosity; and Mb, megabases.

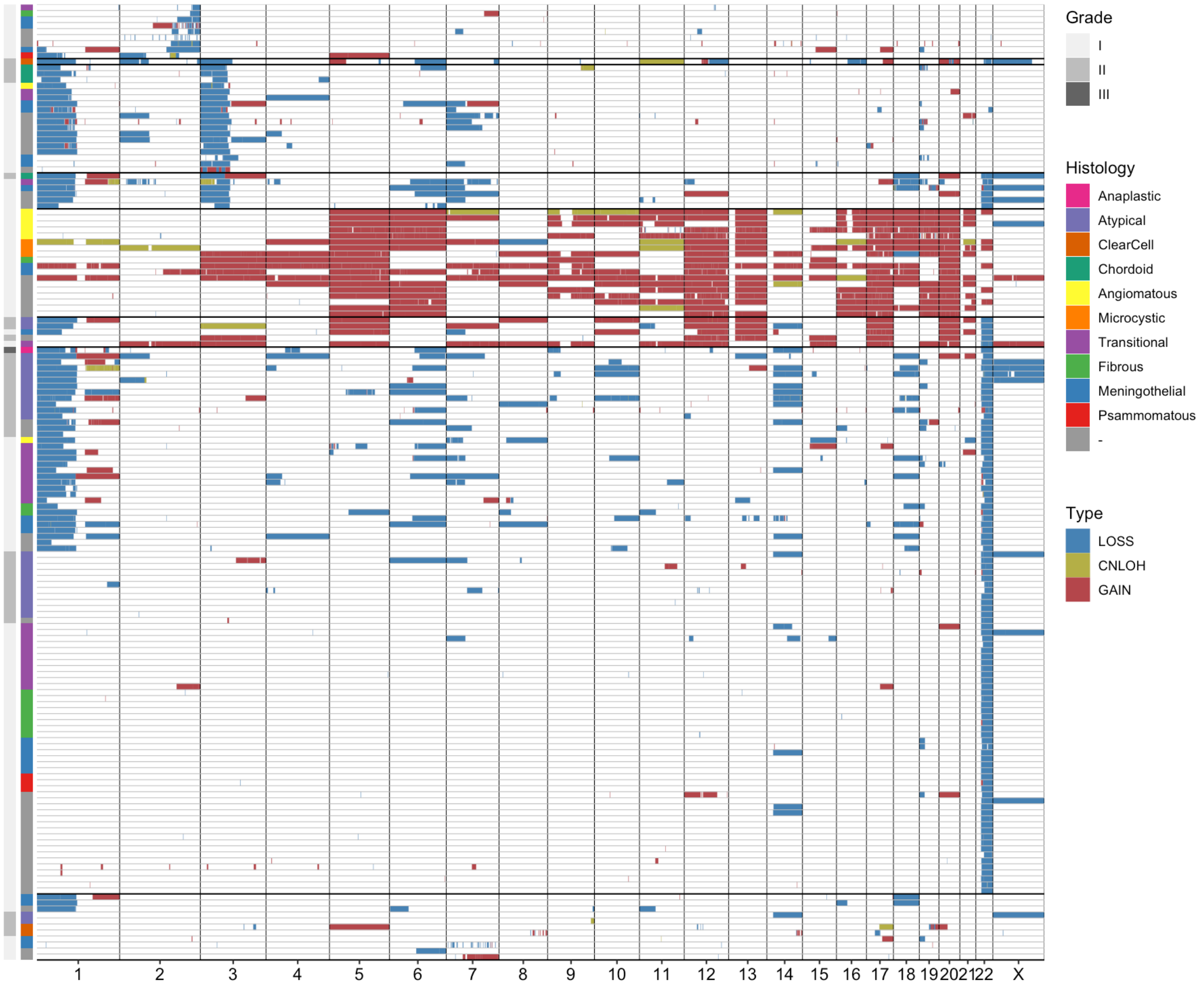

Supplementary Fig. 2: Genomic landscape of SCNAs. A total of 159 samples that acquired at least one large SCNA are shown. Two narrower panels on the left show grade and histology, respectively. The main (right) panel shows locations of SCNAs. Black horizontal lines separate samples according to SCNA driver groups (see main text): (from top) 2q-loss, 2q-loss + 3p-loss + 22q-loss, 3p-loss, 3p-loss + 22q-loss, multiple whole-chromosomal gains, multiple whole-chromosomal gains + 22q-loss, 22q-loss, and others. Source data are provided as a Source Data file with additional information, coverage ratios between tumor and normal samples and the mean deviation of BAF from its overall mean.

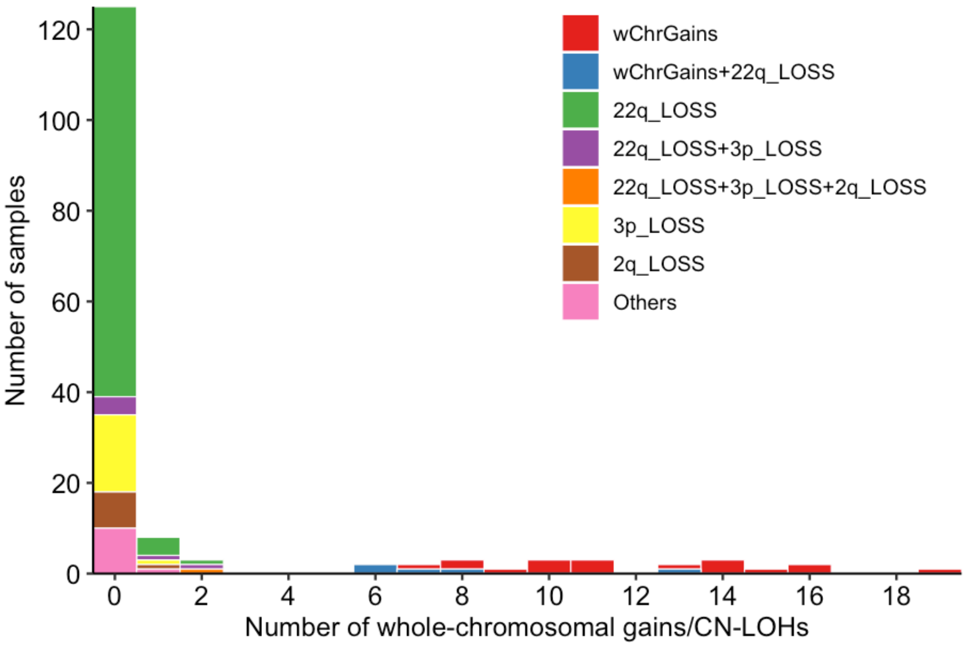

Supplementary Fig. 3: Distribution of the number of whole-chromosomal gains or CN-LOHs among the samples that acquired at least one large SCNA (n = 159). A SCNA is called whole-chromosomal if it covers > 80% of the chromosome. For acrocentric chromosomes, we call a SCNA whole-chromosomal if it covers > 80% of the q-arm. wChrGains, samples with four or more whole-chromosomal gains/CN-LOHs.

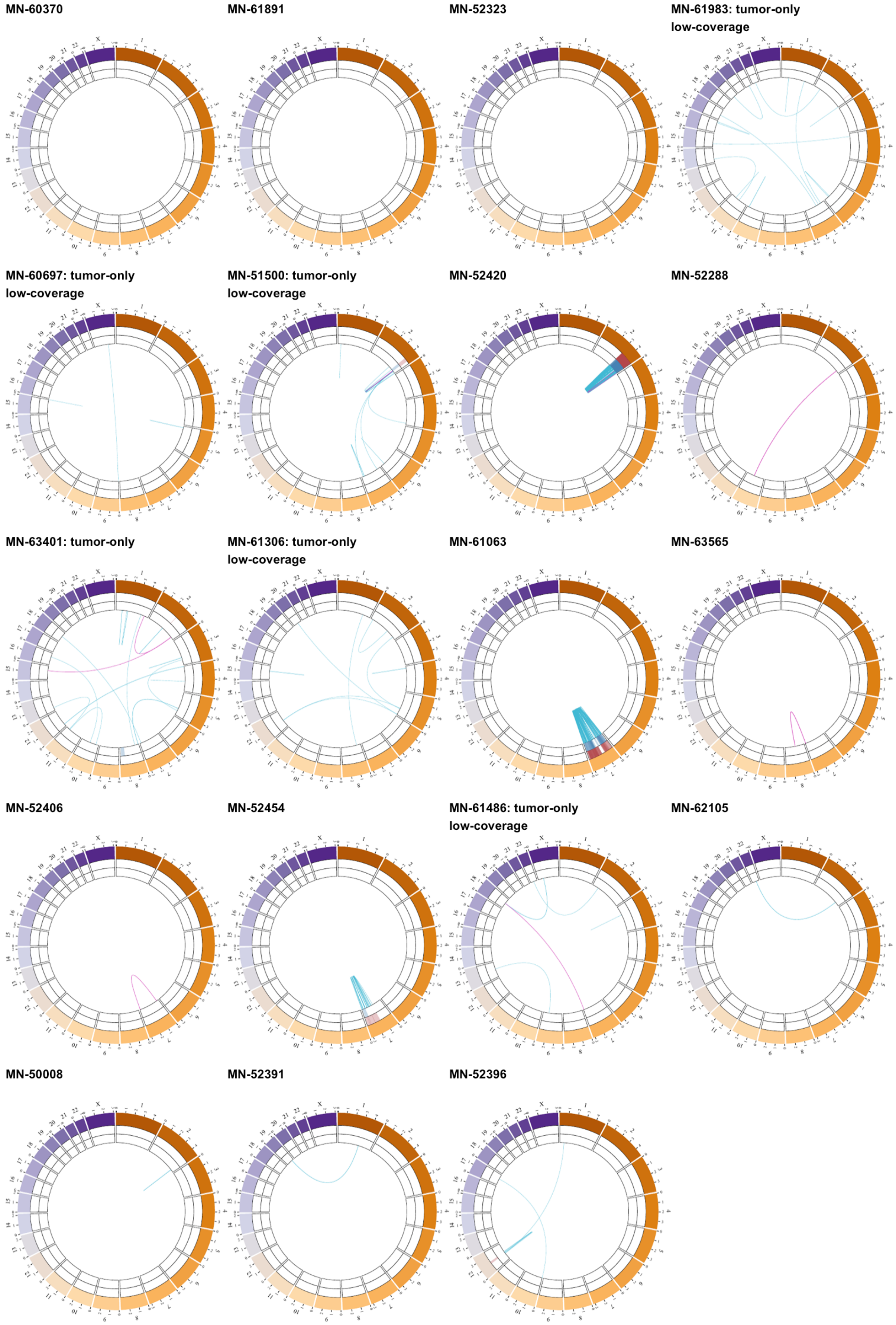
MN-60370
MN-61891
MN-52323
MN-61983: tumor-only low-coverage
MN-60697: tumor-only low-coverage
MN-51500: tumor-only low-coverage
MN-52420
MN-52288
MN-63401: tumor-only
MN-61306: tumor-only low-coverage
MN-61063
MN-63565
MN-52406
MN-52454
MN-61486: tumor-only low-coverage
MN-62105
MN-50008
MN-52391
MN-52396

Supplementary Fig. 4: Structural variations identified from WGS data. The outer track shows duplications, the inner track shows deletions, and the lines show intra- or inter-chromosomal translocations. Translocations with a breakpoint that is close to *IHH* (2:219,925,190-220,100,000) or mapped between *SHH* and *LMBR1* (7:155,604,968-156,461,645) are highlighted with magenta (MN-51500, MN-52420, MN-52288 and MN-63401 for *IHH*; MN-63565, MN-52406 and MN-61486 for *SHH*). MN-61306 and MN-61486 were sequenced to find an event that caused the ectopic expression of *IHH* and *SHH*, respectively, observed in the RNA-Seq analysis (see main text). For tumor-only samples, we expect high false-positive rates even after filtering using a panel of normal samples (Methods).

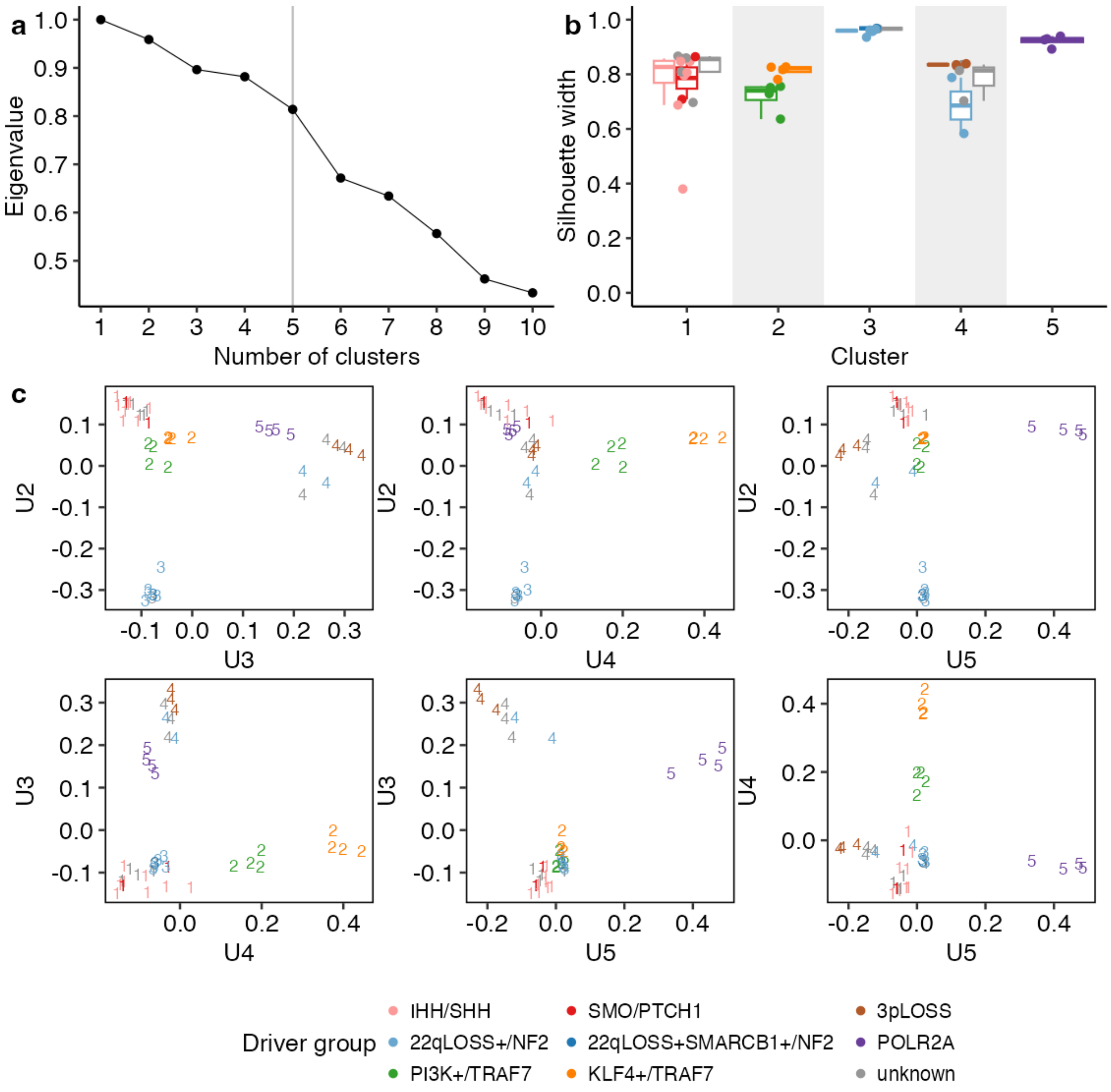

Supplementary Fig. 5: Spectral clustering result of meningiomas using RNA-Seq gene expression data for 42 meningiomas. **a** Eigengap analysis supports a five-cluster model, where the difference of eigenvalues is largest between five- and six-cluster models. **b** Boxplots for the silhouette widths of the cluster membership determined by the Gaussian mixture model. A boxplot indicates median (middle line), the first (Q1) and the third (Q3) quartiles (box), the smallest value down to Q1 – 1.5 IQR and the largest value up to Q3 + 1.5 IQR (whiskers), where IQR = Q3 – Q1. Values beyond the end of the whiskers are plotted individually (outliers). Points are jittered horizontally to avoid overlaps. Within each cluster, data are stratified by driver

groups. **c** The eigenvectors of the normalized adjacency matrix (Uk, k = 2,…,5). The first eigenvector, whose values represent the inverse square-root of the degrees, is omitted. Each sample is shown by its cluster membership.

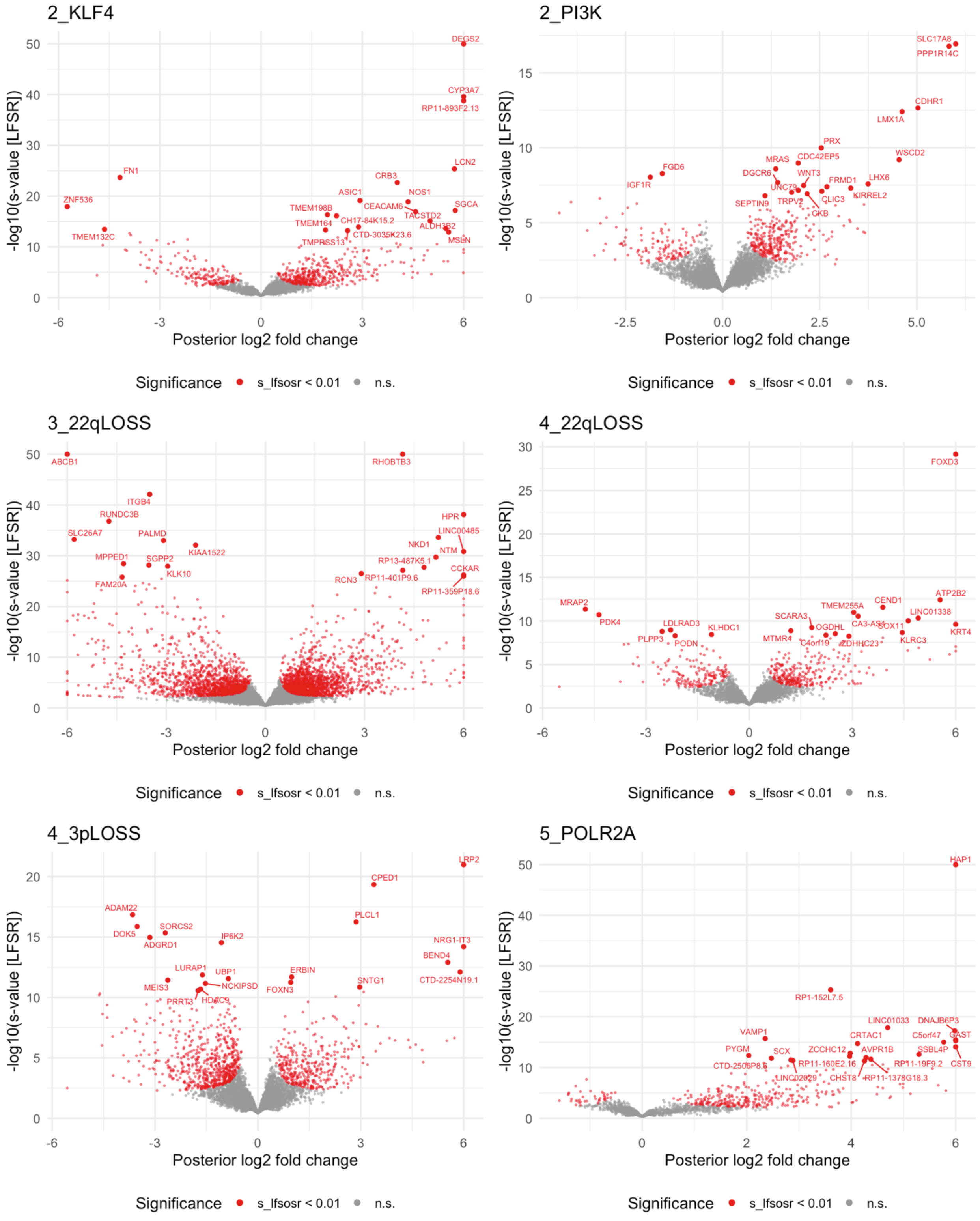
2_KLF4
DEGS2
CYP3A7
RP11-893F2.13
LCN2
CRB3
FN1
NOS1
ASIC1
SGCA
ZNF536
CEACAM6
TACSTD2
TMEM198B
CH17-84K15.2
TMEM164
ALDH3B2
TMEM132C
CTD-3035K23.6
MSLN
TMPRSS13
2_PI3K
SLC17A8
PPP1R14C
CDHR1
LMX1A
PRX
WSCD2
MRAS
CDC42EP5
FGD6
DGCR6
WNT3
LHX6
IGF1R
UNC79
FRMD1
KIRREL2
TRPV2
CLIC3
SEPTIN9
CKB
3_22qLOSS
ABCB1
RHOBTB3
ITGB4
RUNDC3B
HPR
SLC26A7
PALMD
LINC00485
NKD1
KIAA1522
NTM
MPPED1
SGPP2
RP13-487K5.1
KLK10
CCKAR
FAM20A
RCN3
RP11-401P9.6
RP11-359P18.6
4_22qLOSS
FOXD3
ATP2B2
CEND1
TMEM255A
LINC01338
MRAP2
SCARA3
PDK4
LDLRAD3
CA3-AS1
KLHDC1
OGDHL
SOX11
KRT4
PLPP3
MTMR4
KLRC3
PODN
C4orf19
ZDHHC23
4_3pLOSS
LRP2
CPED1
PLCL1
ADAM22
SORCS2
DOK5
IP6K2
NRG1-IT3
ADGRD1
BEND4
LURAP1
UBP1
ERBIN
CTD-2254N19.1
NCKIPSD
MEIS3
FOXN3
SNTG1
PRRT3
HDAC9
5_POLR2A
HAP1
RP1-152L7.5
LINC01033
DNAJB6P3
VAMP1
CRTAC1
C5orf47
CAST
PYGM
SCX
ZCCHC12
AVPR1B
SSBL4P
CTD-2506P8.6
RP11-160E2.16
RP11-19F9.2
CST9
LINC02029
CHST8
RP11-1378G18.3
-log10(s-value [LFSR])
Posterior log2 fold change
Significance
s_lfsosr < 0.01
n.s.

Supplementary Fig. 6: Volcano plots of differential expression analysis result for each transcriptional cluster. Genes that showed s-value based on local false sign or smaller (FSOS) rate < 0.01 at the log2 fold change log2(1.2) are considered significant (colored red, see Methods). The log10 of *s*-values based on the local false sign rate (LFSR) is plotted against the posterior estimates of log2 fold change. The *s*-value was capped at 1×10^{-50} and the posterior log2 fold change was capped at -6 or 6. Top 20 genes ranked by the *s*-value (LFSR) are labeled.

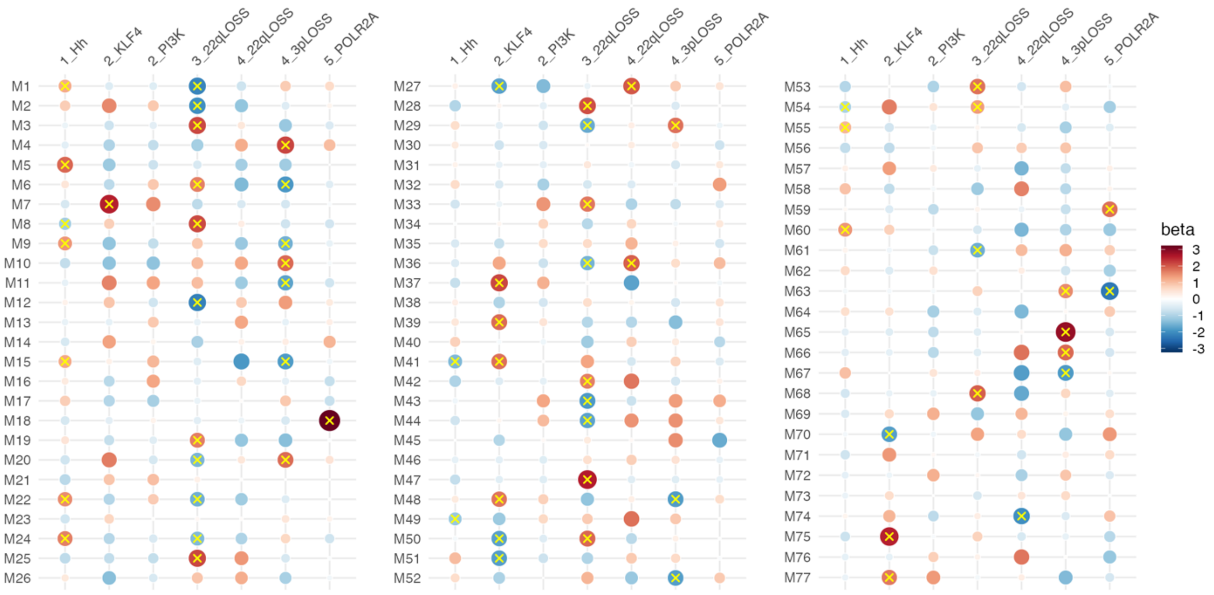

Supplementary Fig. 7: Association of co-expression network modules with transcriptional clusters. The statistical association of seven meningioma subgroups with 77 module eigengenes using linear regression is shown. We found that 51 modules exhibited significant relationships with at least one cluster (after multiple testing correction using Holm's method for 77 modules). The significant associations after multiple testing correction are marked by yellow crosses. The 'beta' refers to the effect size obtained from the linear regression and is represented by the size and color gradient. As we aligned the module eigengene such that the positive values correspond to increased expressions, we can interpret those genes in the module are upregulated when the estimated beta is positive. See also Supplementary Data 14.

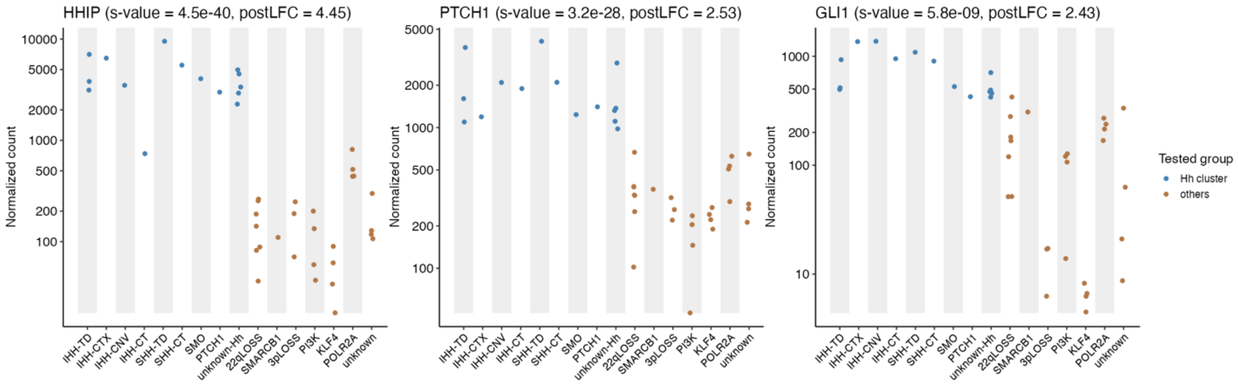

Supplementary Fig. 8: Upregulation of Hh signaling target molecules in the Hh cluster members. These Hh signaling markers are significantly upregulated in meningiomas of the Hh cluster consistently regardless of the types of mutations and structural variations. *HHIP* and *PTCH1* are the top two most significantly DE genes for the Hh cluster (Fig. 3b and Supplementary Data 12). The size factor-normalized read count is shown on the vertical axis while the horizontal axis is stratified by the genomic driver event. Samples are colored according to gene expression cluster (Hh vs. others). On top of each panel, *s*-value (based on LFSR) and the posterior log2 fold change (postLFC) from DE analysis are shown. TD, tandem duplication. CTX, inter-chromosomal translocation. CNV, copy number variation. CT, chromothripsis.

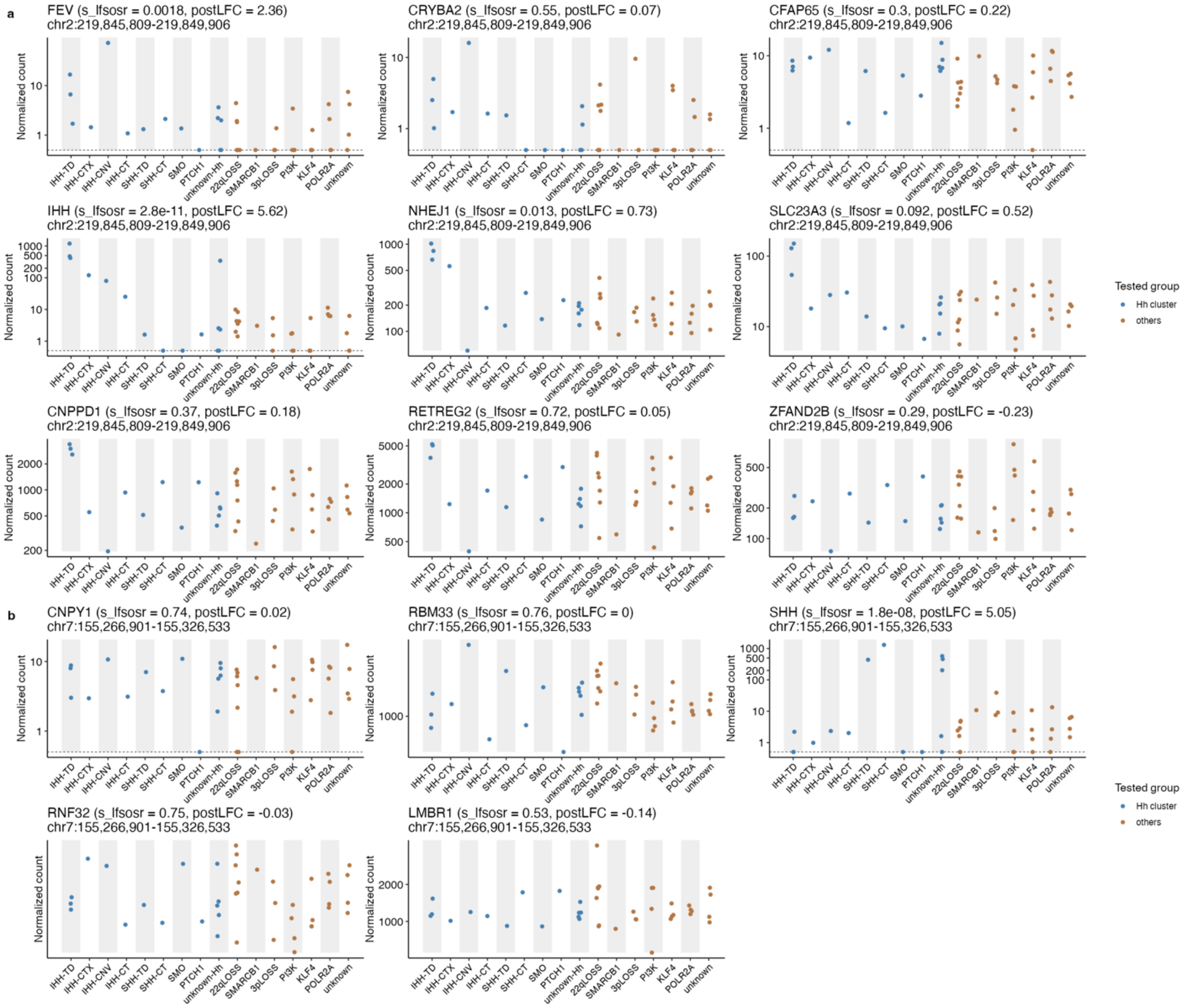

Supplementary Fig. 9: Ectopic expression of genes in 2q35. The size factor-normalized read count is shown on the vertical axis while the horizontal axis is stratified by the genomic driver event. Samples are colored according to gene expression cluster (Hh vs. others). On top of each panel, we show *s*-value (based on local false sign or smaller rate [LFSOSR]) for the significance (significant DE gene if *s*_lfsosr < 0.01) and the posterior log2 fold change (postLFC) for the effect size from DE analysis, as well as the gene coordinate (in Hg19). **a** It is notable that among the four genes (*FEV*, *CRYBA2*, *CFAP65*, *IHH*) where the copy numbers were preserved or gained (Fig. 2b), only *IHH* showed a consistent upregulation of all the samples that acquired 2q35 events (and one sample that belongs to the Hh cluster without identified driver). Genes telomeric to *IHH* also show ectopic expression in tandem duplication cases (IHH-TD) and an inter-chromosomal translocation case (IHH-CTX, MN-52288). For the latter, the breakpoint at 2q35 is located within *SLC23A3* (so this gene is not upregulated for this sample). All the tandem

duplication cases acquired a breakpoint between *RETREG2* and *ZFAND2B*. **b** All the meningiomas that acquired structural variations on 7q (as well as three samples without identified driver) are consistently upregulated at *SHH*, while genes flanking SHH are not co-upregulated with *SHH*. TD, tandem duplication. CTX, inter-chromosomal translocation. CNV, copy number variation. CT, chromothripsis.

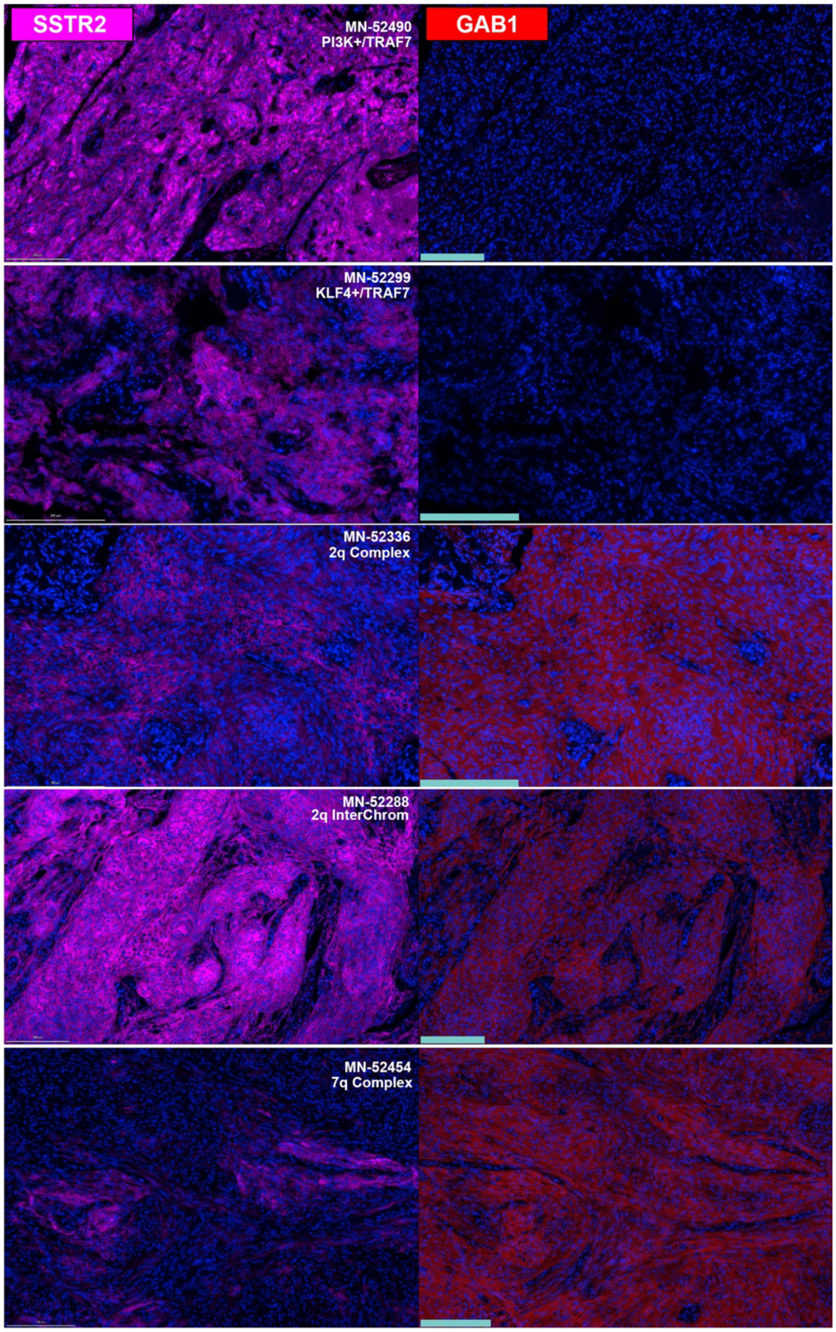

Supplementary Fig. 10: Additional Multiplexed Immunofluorescence Images. As in **Fig. 4**, staining for the meningioma marker SSTR2 and Hh activation marker GAB1 are shown. The light blue bar represents 200um. On each row two images are shown from the same patient (5 total patients).

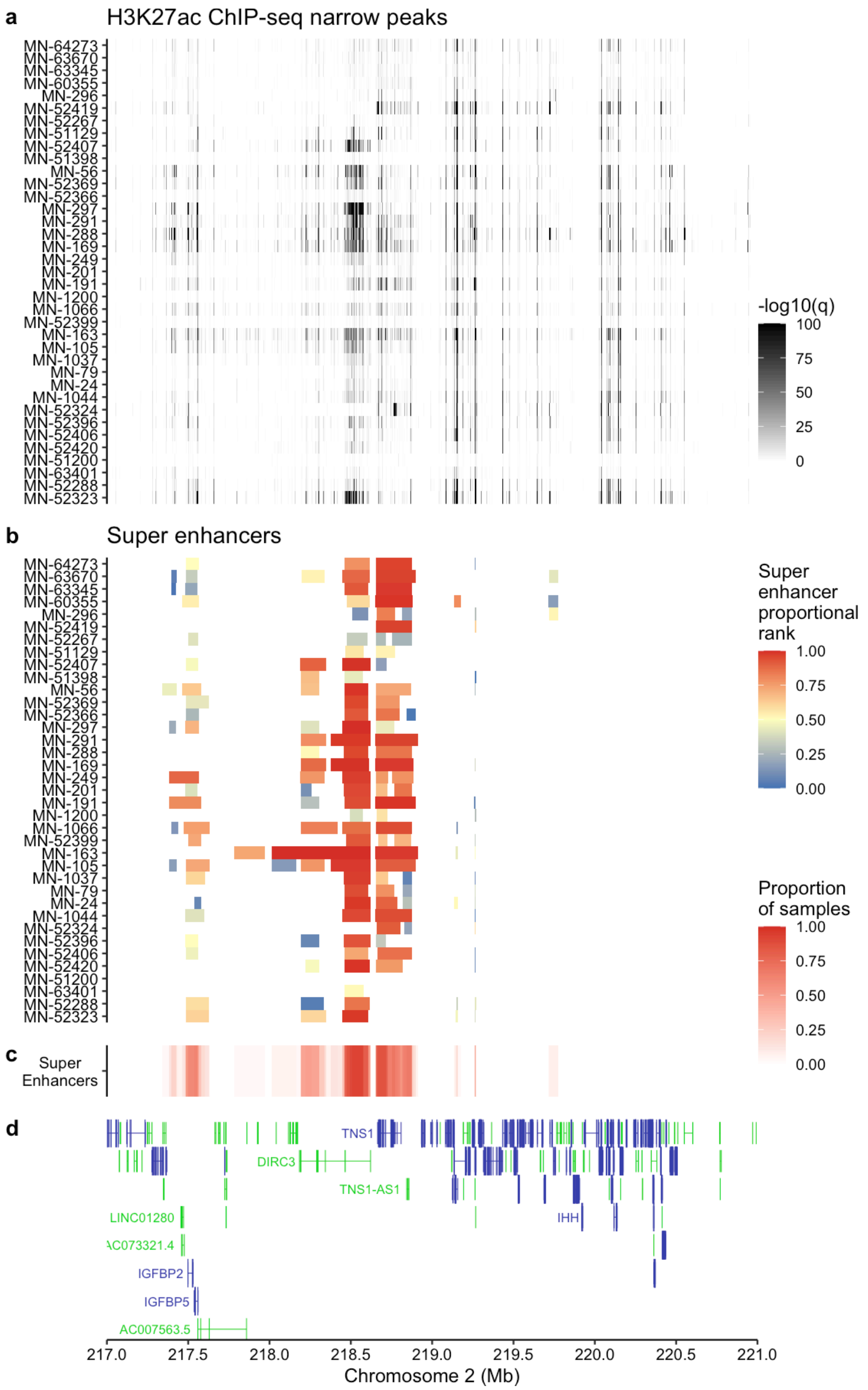

Supplementary Fig. 11: Super-enhancers at 2q35. a H3K27ac ChIP-seq narrow peaks detected by MACS2. Darker colors indicate more significant (in terms of the *q*-value) peaks. The *q*-values were capped at 1×10^{-100}. There is no significant H3K27ac signal near the *IHH* locus itself, consistent with an absence of active proximal enhancers. **b** Super-enhancers detected by ROSE. Colors were assigned based on the proportional rank (top = 1.0, the super-enhancer

closest to the typical enhancer = 0). **c** The proportion of samples with overlapping super-enhancers. **d** Gencode genes (see the legend of Fig. 2).

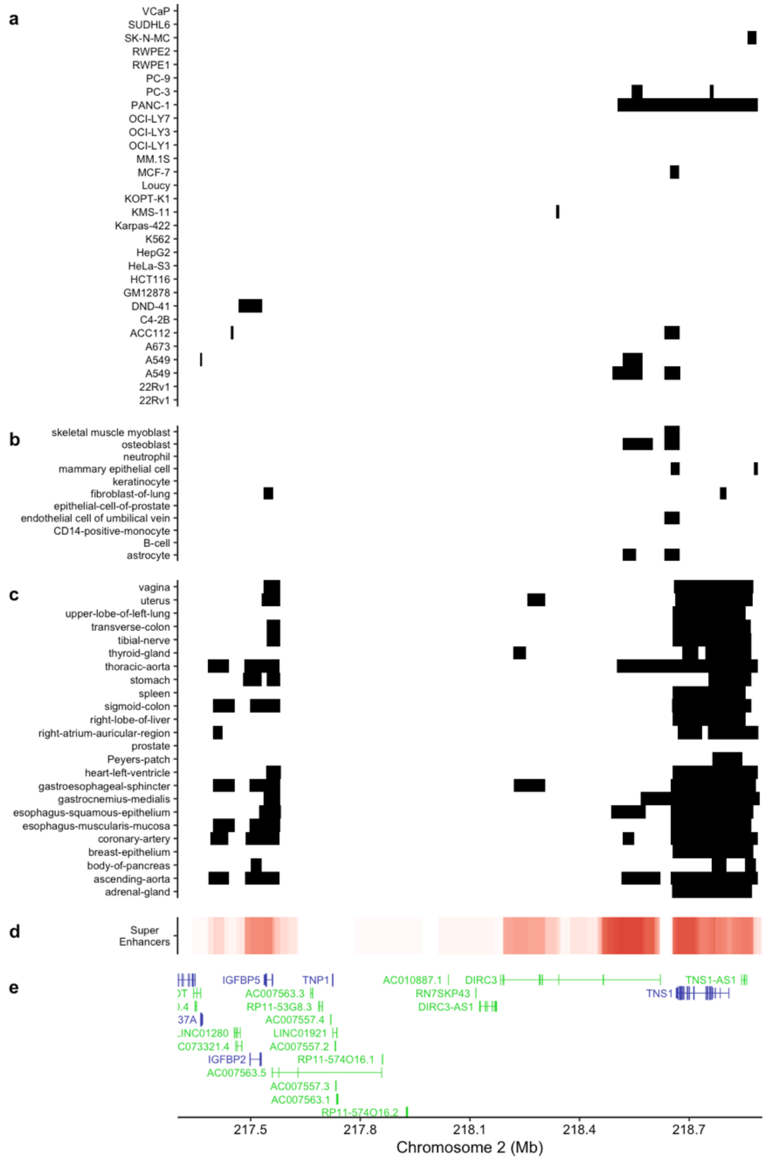

Supplementary Fig. 12: Super-enhancers in ENCODE samples from 217.3 Mb to 218.9 Mb on chromosome 2. a Cell cultures. **b** Primary cells. **c** Tissues. **d** The proportion of meningioma samples with overlapping super-enhancers (the color scale is the same as the panel **c** of Supplementary Fig. 11). **e** Gencode genes (see the legend of Fig. 2). Protein-coding genes are shown in blue. For each gene, only the canonical transcript is selected. *DIRC3* super-enhancers observed commonly in meningiomas (panel **d**, see also Supplementary Fig. 11) are relatively tissue-specific compared to those overlapping *IGFBP2* and *TNS1*. The ENCODE super-enhancer data was downloaded from https://sunlightwang.github.io/Super-Enhancers/.

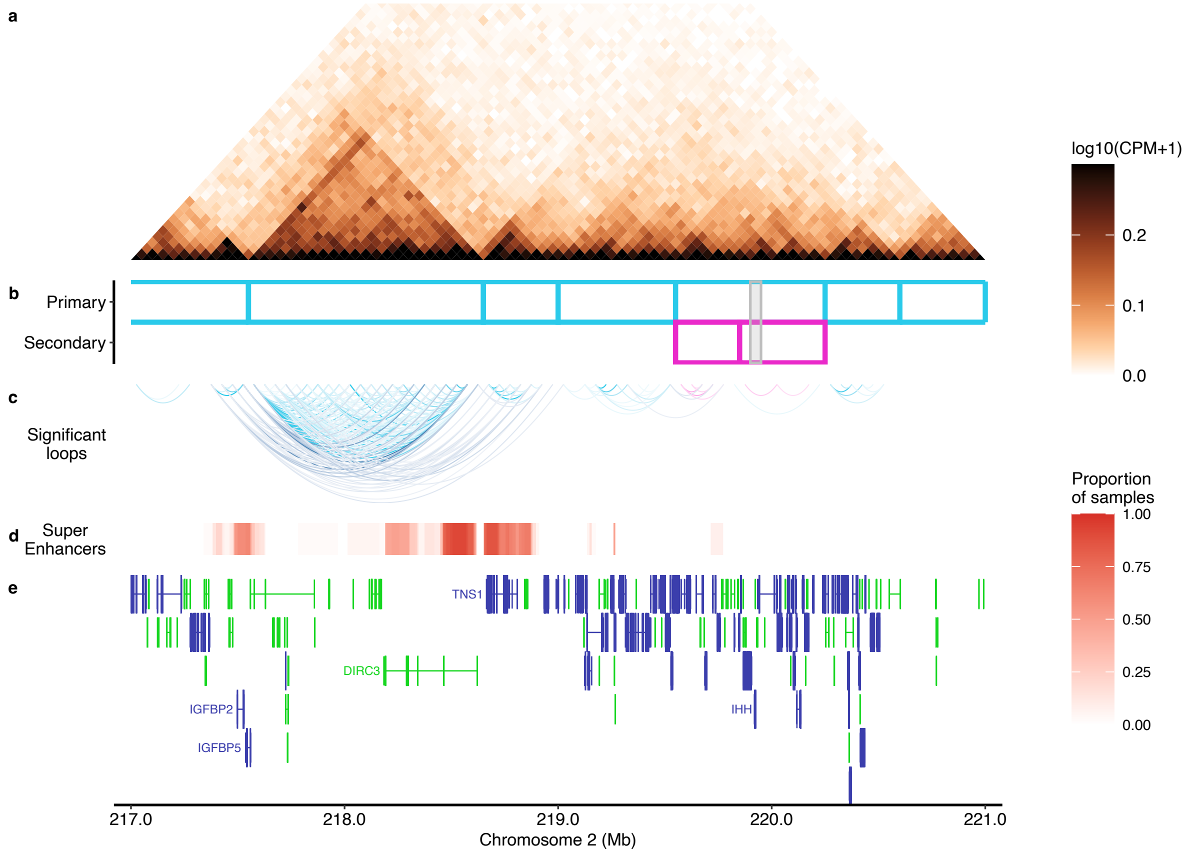

Supplementary Fig. 13: HiChIP analysis result for control set. HiChIP data of control meningiomas revealed minimal chromatin interactions involving the IHH locus. Super-enhancer regions found within *DIRC3* belong to a distinct TAD, sequestered from interacting with genes in the TAD that contained *IHH*. **a** HiChIP map, colors are based on the logarithm (base 10) of counts per million (CPM). **b** TAD boundaries detected by SpectralTAD. A 50 kb interval that contains *IHH* is represented by a gray rectangle. **c** Significant loops identified by FitHiChIP. Within-TAD loops are depicted by the same colors as TAD boundaries while loops that exceed TAD boundaries are shown by steel blue curves. **d** Proportion of meningiomas in our H3K27ac ChIP-seq cohort (n = 37) that harbor an overlapping super-enhancer in the designated region. **e** Gencode basic genes (v38lift37) in this region. Only genes overlapping the common super-enhancers as well as *IHH* are labeled.

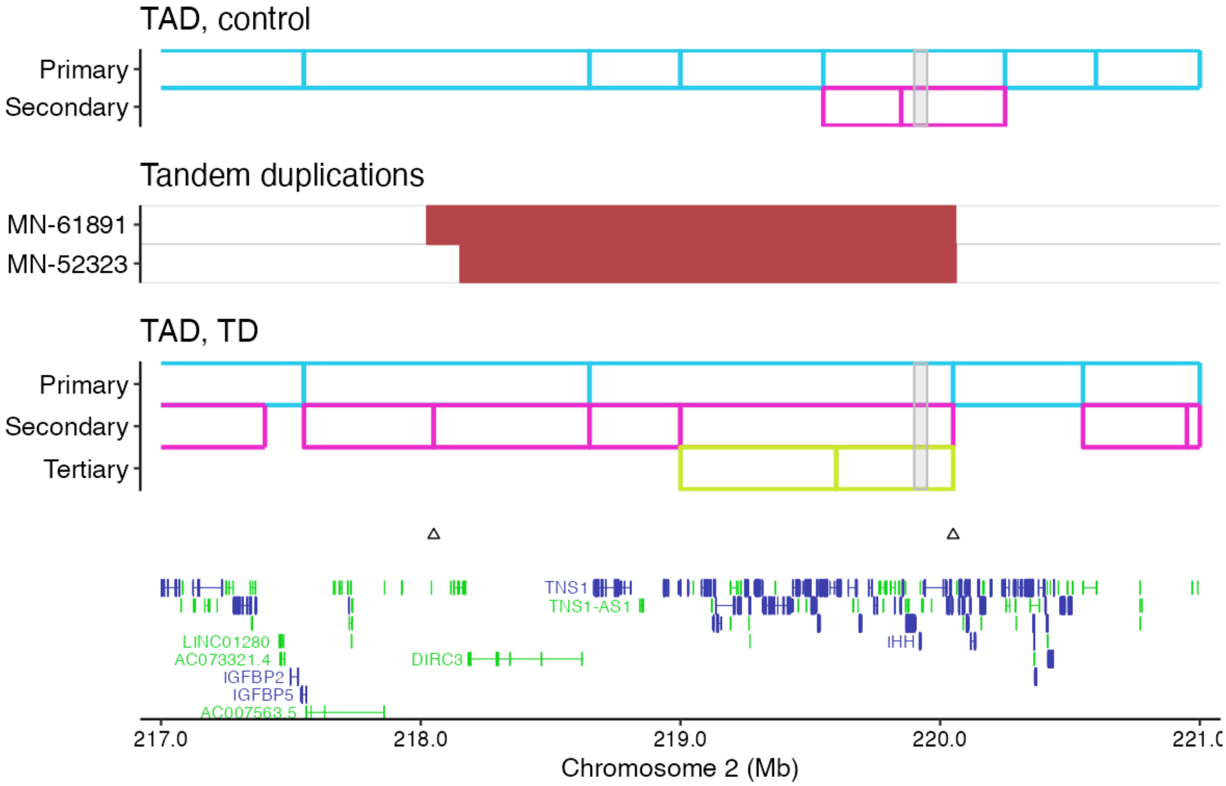

Supplementary Fig. 14: Alteration of TAD structure due to tandem duplication. Top and the third panels show TAD boundaries detected by Spectral TAD for the control and TD set, respectively. The second panel shows the tandem duplications observed in the two samples included in the TD set. In the TD set, two novel TAD boundaries emerged at 218.05 Mb and 220.05 Mb (marked by triangles), associated with the tandem duplication breakpoints. A 50 kb bin for the HiChIP analysis that includes *IHH* (from 219.90 Mb to 219.95 Mb) is shown as a gray rectangle in the top and the third panel.

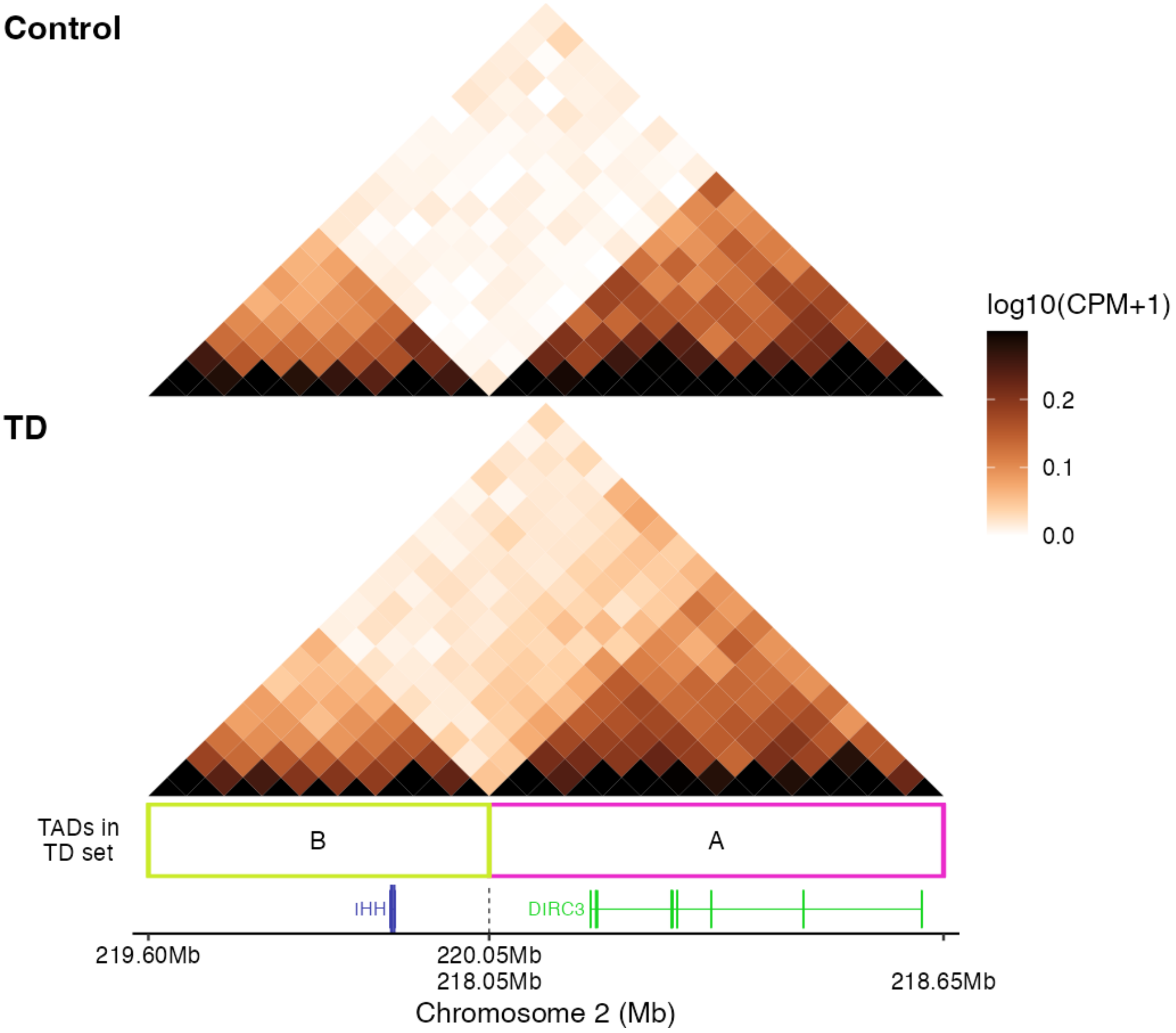

Supplementary Fig. 15: Neo-TAD formed by tandem duplication involving *IHH*. We extracted two segments corresponding to the two TADs found in tandem duplication cases labeled A and B in Fig. 5, which located in the boundaries of tandem duplication, and mapped them next to each other. The top interaction plot (Control) depicts the same region in the control set, which demonstrates that two regions do not interact significantly. The second plot (TD) depicts the region in the TD set, which demonstrates significant interaction between A and B. Below, the two distant TADs in Fig. 5, which are now in the neighbor as a result of tandem duplication, as well as genes of interest are drawn.

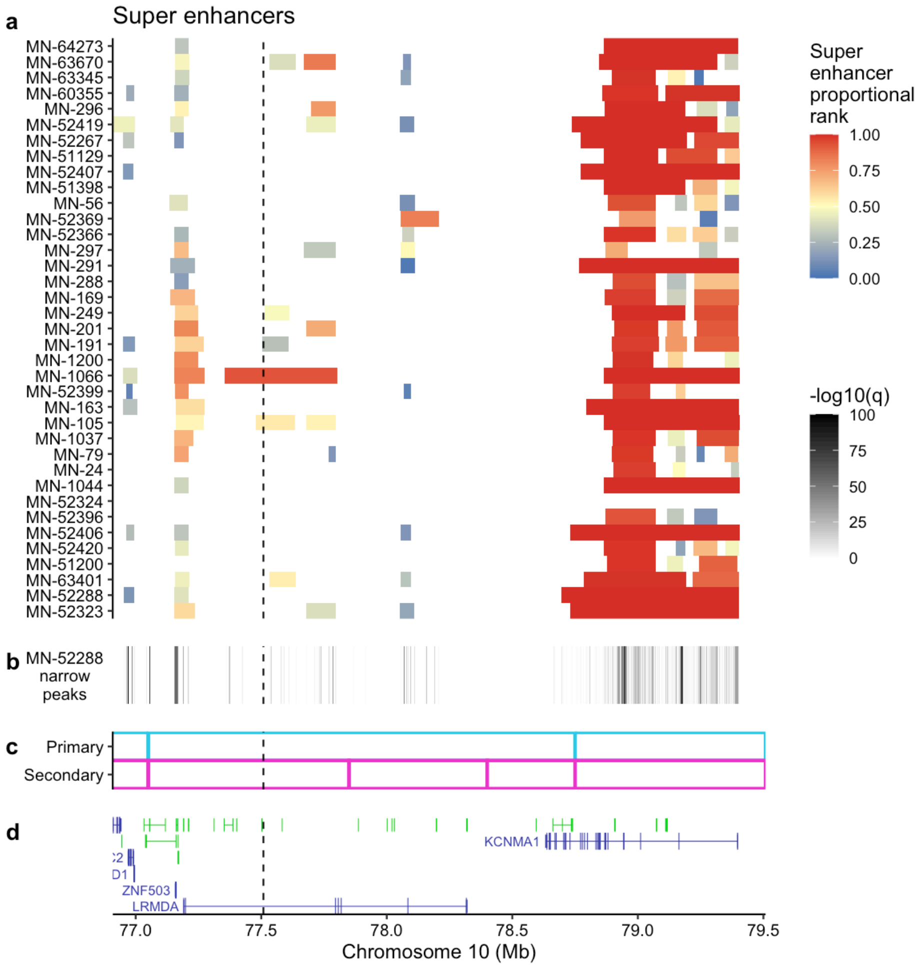

Supplementary Fig. 16: Chromosome 10 segment (10:77,508,708-qter) juxtaposed to near *IHH* (2:1-220,029,008) in MN-52288. The dashed line shows the breakpoint and the segment to the right of it is juxtaposed to chromosome 2 that contains *IHH*. **a** Super-enhancers in the meningioma cohort. Colors were assigned based on the proportional rank (top = 1.0, the super-enhancer closest to the typical enhancer = 0). **b** Narrow peaks detected by MAC2 for MN-52288. Darker colors indicate more significant (in terms of the q-value) peaks. The q-values were capped at 1×10^{-100}. **c** TAD boundaries detected by SpectralTAD for the control set. **d** Gencode genes (see the legend of Fig. 2).

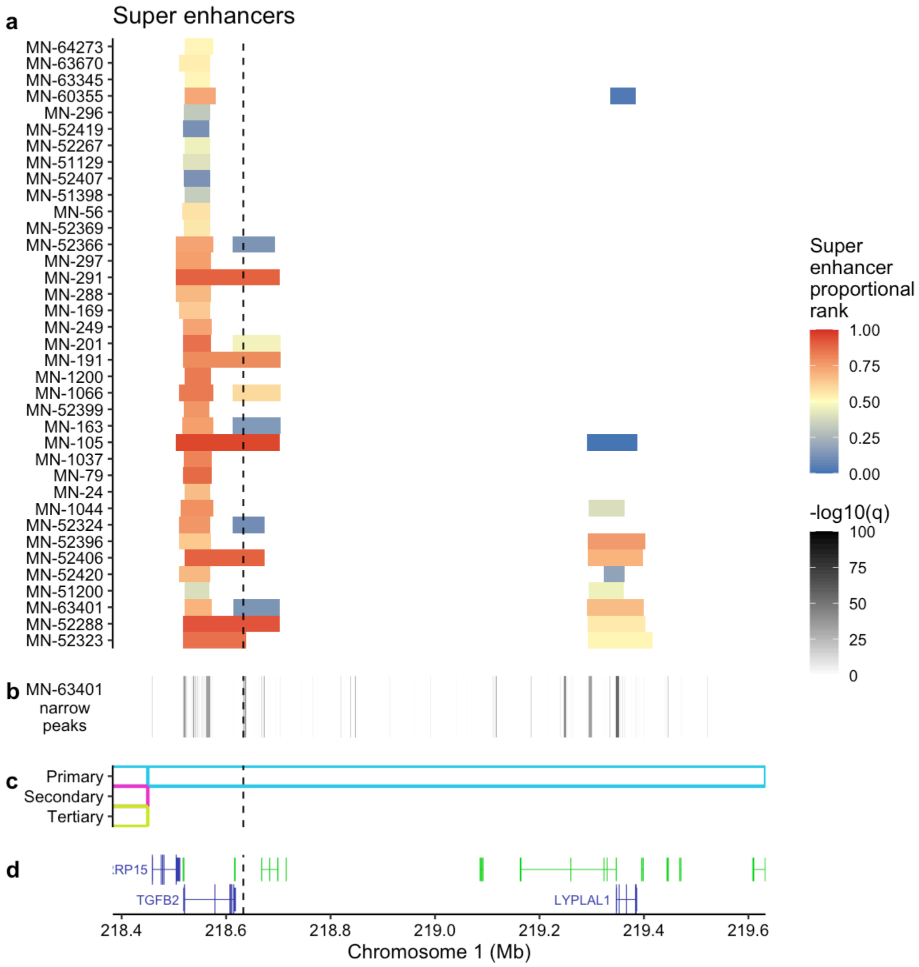

Supplementary Fig. 17: Chromosome 1 segment (1:218,632,910-qter) juxtaposed to near IHH (2:1-220,034,453) in MN-63401. The dashed line shows the breakpoint and the segment to the right of it is juxtaposed to chromosome 2 that contains *IHH*. **a** Super-enhancers in the meningioma cohort. Colors were assigned based on the proportional rank (top = 1.0, the super-enhancer closest to the typical enhancer = 0). **b** Narrow peaks detected by MAC2 for MN-63401. Darker colors indicate more significant (in terms of the q-value) peaks. The q-values were capped at 1×10^{-100}. **c** TAD boundaries detected by SpectralTAD for the control set. **d** Gencode genes (see the legend of Fig. 2).

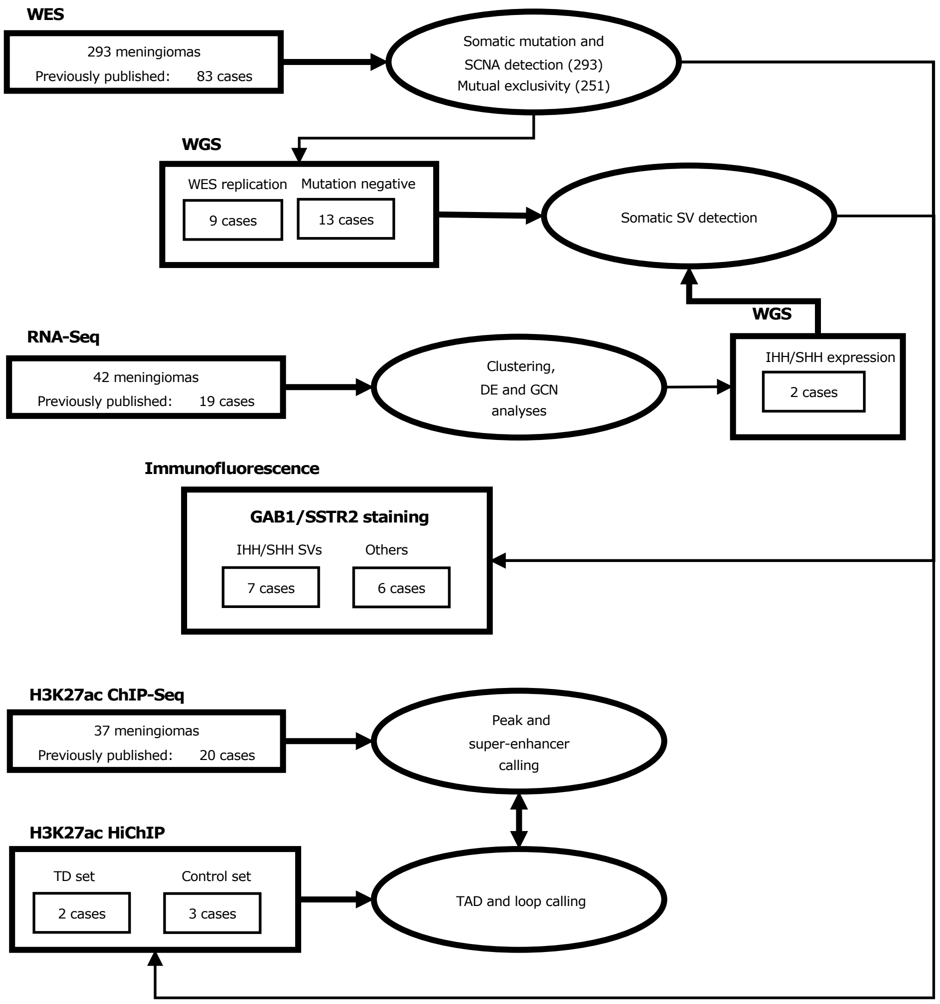

Supplementary Fig. 18: Study design overview. Genomic, transcriptomic and epigenomic data were analyzed to identify previously unknown somatic alterations associated with meningioma. Some analyses included datasets previously published by our lab, as indicated by “Previously published” (see also Supplementary Data 1; PMIDs 27548314 and 28195122). For the GAB1/SSTR2 staining experiments, results of four cases of ‘IHH/SHH SVs’ and the same number of ‘Others’ are presented in Fig. 4, while the remaining samples are presented in Supplementary Fig. 10.

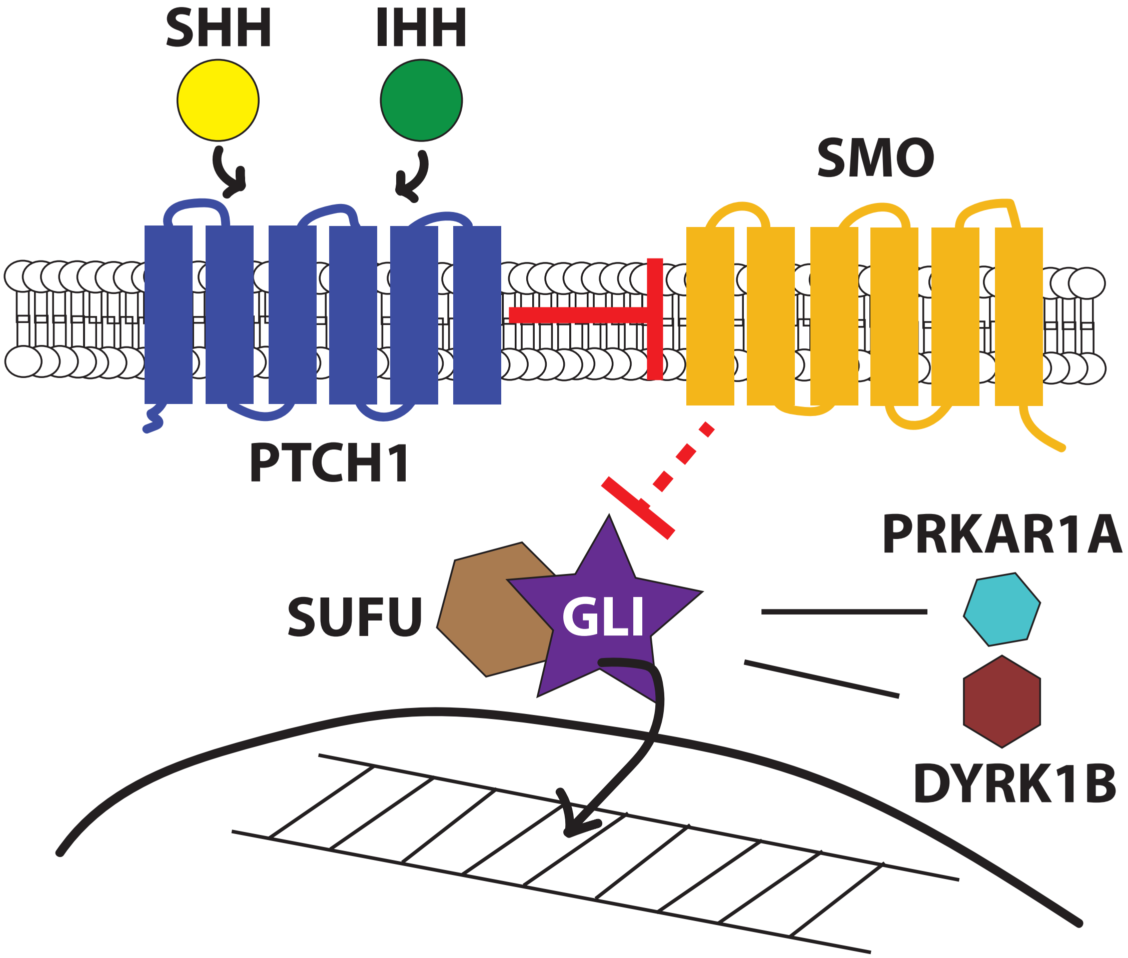

Supplementary Fig. 19: Hh Pathway Genes Involved in Meningiomas. Shown are Hh pathway molecules that are implicated in meningiomas. Binding of the ligands SHH or IHH to PTCH1 releases its constitutive inhibition of SMO, resulting in translocation of the transcription factor GLI1 to the nucleus to activate Hh pathway transcriptional targets. SUFU is a negative regulator of Hh that blocks translocation of GLI1. DYRK1B and PRKAR1A are regulators of GLI signaling.
