# Supplementary material for: Super-enhancer hijacking drives ectopic expression of hedgehog pathway ligands in meningiomas
Source: Nat Commun. 2023 Oct 7;14:6279. doi: 10.1038/s41467-023-41926-y (PMC10560290; doi:10.1038/s41467-023-41926-y)
Supplement: Supplementary file 2 — Description of Additional Supplementary Files [file 41467_2023_41926_MOESM2_ESM.pdf]

Description of Additional Supplementary Files

File Name: Supplementary Data 1
Description: Sample information

File Name: Supplementary Data 2
Description: Frequencies of large SCNA events

File Name: Supplementary Data 3
Description: Mutually exclusive event pairs

File Name: Supplementary Data 4
Description: Co-occurring event pairs.

File Name: Supplementary Data 5
Description: Clinical characteristics of meningiomas with 3p-loss

File Name: Supplementary Data 6
Description: Clinical characteristics of meningiomas with 2q complex rearrangements

File Name: Supplementary Data 7
Description: Focal gains at 2q35

File Name: Supplementary Data 8
Description: Clinical characteristics of meningiomas with focal gain at 2q35.

File Name: Supplementary Data 9
Description: SCNAs on 7q observed in driver-unknown meningiomas.

File Name: Supplementary Data 10
Description: Clinical characteristics of meningiomas with SCNAs on 7q.

File Name: Supplementary Data 11
Description: Structural variations detected by Manta.

File Name: Supplementary Data 12
Description: Differentially expressed genes in each transcriptional cluster

File Name: Supplementary Data 13
Description: Genes in the gene co-expression network modules

File Name: Supplementary Data 14
Description: Association between transcriptional clusters and module eigengene

File Name: Supplementary Data 15
Description: Module characterization

File Name: Supplementary Data 16

Description: Gene set overrepresentation analysis result

File Name: Supplementary Data 17
Description: Module assignment of top 30 differentially expressed genes for each transcriptional cluster

File Name: Supplementary Data 18
Description: Significant loops identified by FitHiChIP in TD set (from 217 Mb to 221 Mb on chromosome 2).
