# Supplementary material for: Super-enhancer hijacking drives ectopic expression of hedgehog pathway ligands in meningiomas
Source: Nat Commun. 2023 Oct 7;14:6279. doi: 10.1038/s41467-023-41926-y (PMC10560290; doi:10.1038/s41467-023-41926-y)
Supplement: Supplementary file 21 — Reporting Summary [file 41467_2023_41926_MOESM21_ESM.pdf]

nature portfolio

Corresponding author(s): Murat Gunel; Katsuhito Yasuno

Last updated by author(s): Sep 22, 2023

# Reporting Summary

Nature Portfolio wishes to improve the reproducibility of the work that we publish. This form provides structure for consistency and transparency in reporting. For further information on Nature Portfolio policies, see our Editorial Policies and the Editorial Policy Checklist.

## Statistics

For all statistical analyses, confirm that the following items are present in the figure legend, table legend, main text, or Methods section.

| n/a | Confirmed | |
|---|---|---|
| ☐ | ☒ | The exact sample size (*n*) for each experimental group/condition, given as a discrete number and unit of measurement |
| ☐ | ☒ | A statement on whether measurements were taken from distinct samples or whether the same sample was measured repeatedly |
| ☐ | ☒ | The statistical test(s) used AND whether they are one- or two-sided<br>*Only common tests should be described solely by name; describe more complex techniques in the Methods section.* |
| ☐ | ☒ | A description of all covariates tested |
| ☐ | ☒ | A description of any assumptions or corrections, such as tests of normality and adjustment for multiple comparisons |
| ☐ | ☒ | A full description of the statistical parameters including central tendency (e.g. means) or other basic estimates (e.g. regression coefficient) AND variation (e.g. standard deviation) or associated estimates of uncertainty (e.g. confidence intervals) |
| ☐ | ☒ | For null hypothesis testing, the test statistic (e.g. *F*, *t*, *r*) with confidence intervals, effect sizes, degrees of freedom and *P* value noted<br>*Give P values as exact values whenever suitable.* |
| ☒ | ☐ | For Bayesian analysis, information on the choice of priors and Markov chain Monte Carlo settings |
| ☒ | ☐ | For hierarchical and complex designs, identification of the appropriate level for tests and full reporting of outcomes |
| ☐ | ☒ | Estimates of effect sizes (e.g. Cohen's *d*, Pearson's *r*), indicating how they were calculated |

*Our web collection on statistics for biologists contains articles on many of the points above.*

## Software and code

Policy information about availability of computer code

Data collection

Whole-exome sequencing data was acquired for samples listed in supplementary table 1. The SeqCap EZ Exome v2.0 (Roche Life Science), xGen Exome Research Panel (Integrated DNA Technologies), or SeqCap EZ MedExome (Roche Life Science) kit was used for capture. After library preparation, sequencing was performed on the Illumina HiSeq platform (RRIDs: SCR_016386, SCR_016383, SCR_016387) with paired-end 74 or 100 base pair reads. The tumor and blood samples were sequenced to a target depth of 185 and 85 reads, respectively. Sequencing data was collected on the Illumina platform, using manufacturer provided software.

Meningiomas that lacked known drivers, as well as driver SCNAs identified in this paper, or some of those that acquired SCNAs at 2q35 or 7q36.3 in the WES analysis, were selected for whole-genome sequencing (WGS). WGS libraries were prepared using the Illumina TruSeq DNA PCR-Free sample preparation kit (Illumina, FC-121-3001) according to the manufacturer's protocol. Data were acquired on the NovoSeq platform (Illumina), using paired-end, 150 base pair read. The target coverage depth for tumor and blood samples was 60x and 30x, respectively. Five of the tumor-only samples were targeted for 20x as they acquired SCNAs at 2q35 or clustered to the Hedgehog sample cluster in RNA-Seq data.

RNA-sequencing (RNA-Seq) analysis was performed on a cohort of 42 meningiomas (19 previously reported). The RNA Integrity Number (RIN) was determined using a Bioanalyzer 2100 (Agilent; RRID:SCR_019389). Each specimen underwent ribozero depletion and was sequenced on the Illumina HiSeq platform (RRIDs: SCR_016386, SCR_016383, SCR_016387) using paired-end, 75 base pair read.

Automated multiplexed sequential Immunofluorescence (seqIF™) imaging was performed on FFPE meningioma slides using the COMET™ platform (Lunaphore Technologies). The multiplex panel included antibodies for SSRTR2 (Ab134152; 1/2000; Rabbit), a meningioma marker, and GAB1 (Cell signaling Polyclonal 3232; 1/100; Rabbit), a marker for Hh pathway activation. All antibodies were validated using conventional

IHC and/or IF staining in conjunction with corresponding fluorophores and 4',6-diamidino-2-pheynlindole counterstain (DAPI, ThermoFisher Scientific).

For ChIP-seq, frozen tumor specimens were sectioned into 15 μM slices and immediately cross-linked with 1% formaldehyde for 15 minutes. Samples were quenched with glycine for 10 minutes, then washed twice with chilled Phosphate Buffered Saline (PBS). Tissues were disrupted using dounce homogenization, then re-suspended in nuclear lysis buffer with 0.2% Sodium Dodecyl Sulfate (SDS). Samples were then sheared to achieve fragments of 100-300 base pairs using a QSonica Q800R sonicator (amplitude 70%). Chromatin was incubated overnight with beads coated in H3K27ac antibody (Abcam ab4729, RRID:AB_2118291), while 10% of the sample was saved as input for later sequencing. The next day, beads were washed with low and high salt buffers, then chromatin was eluted, and crosslinks were reversed. Samples were purified using the Qiagen PCR purification kit. Sequencing was performed on the Illumina HiSeq platform using single-end 75 base pair reads. Replicates for each genomic subgroup were included when available, and each replicate represented a distinct patient tumor specimen.

For H3K27ac Hi-ChIP, 15 to 30 slices (20 μm) of each flash-frozen tumor specimen were sectioned using a cryostat and collected in chilled PBS. Samples underwent dounce homogenization, then fixation in 1% formaldehyde for 15 minutes. The reaction was quenched in glycine for 10 minutes, then washed twice with chilled PBS. Pelleted samples were flash frozen in liquid nitrogen and stored for later use. For HiChIP, pellets were thawed on ice and incubated in Hi-C lysis buffer for 30 minutes with rotation. Nuclei were pelleted and washed again with Hi-C lysis buffer, then incubated in 0.5% SDS for 10 minutes at 62 degrees. The reaction was quenched in Triton X-100 for 15 minutes at 37 degrees. Samples were then incubated with MboI restriction enzyme (NEB, R0147) for two hours. MboI was heat-inactivated for 20 minutes (62 degrees), followed by extension of overhands using biotin-ATP (Thermo Fisher, 19524016) for 1 hour. Fragments were ligated using T4 DNA ligase (NEB, M0202) for 4 hours. Samples were then pelleted and re-suspended in Nuclear Lysis Buffer. ChIP-seq was then performed as described above, using an amplitude of 70% and duration of 10 minutes. After sonication, samples were diluted to reduce SDS concentration, then incubated with H3K27ac antibody (Abcam ab4729, RRID:AB_2118291) overnight (5 μg per sample). The next morning, 50 μL of washed beads were added to each tube and incubated for 2 hours with rotation (4 degrees). Samples were washed with low-salt, high-salt, and LiCl buffer 3 times each at room temperature on the magnet. DNA was eluted then purified with DNA Clean and Concentrator columns (Zymo Research). Streptavidin C1 beads (Thermo Fisher) were washed with Tween Wash Buffer and resuspended in 2x Biotin Binding Buffer, then 5 μL of beads was added to each tube. Samples were incubated for 15 minutes shaking, then washed twice with Tween Wash Buffer. They were incubated at 55 degrees with shaking during each wash. Subsequently, samples were washed with 1x Tagment DNA (TD) buffer (Illumina), then re-suspended for the Tn5 transposase reaction. A total of 0.2 μL Tn5 was used for each sample, and they were incubated at 55 degrees with shaking for 10 minutes. Samples were then incubated with 50 mM EDTA for 30 minutes at 50 degrees, then two times each with 50 mM EDTA and Tween Wash Buffer. Samples were then washed with 10 mM Tris and underwent 10 rounds of PCR amplification using Nextera primers. After PCR, samples were placed on the magnet and the supernatant was collected and purified (Zymo Research). DNA was quantified using picogreen and balanced between samples prior to pooling.

Available medical records were reviewed to generate the patient information in supplementary table 1.

Data analysis

For whole-exome sequencing data, sequenced reads were aligned to the human reference genome (GRCh37) using BWA-mem (version 0.7.15). PCR duplicates were marked with Picard (version 2.17.11, RRID:SCR_006525), followed by local realignment and base quality recalibration using Genome Analysis Toolkit (GATK, version 3.4, RRID:SCR_001876). Somatic Copy Number Alterations (SCNAs) were inferred with the ExomeCNV R package (RRID:SCR_010815), using the logarithm (base 2) of the coverage ratios (logCR). See methods section for additional details, including merging of segments and filtering. Discrete Independence Statistic Controlling for Observations with Varying Event Rates (DISCOVER) was used to determine co-occurrence of large SCNAs. Somatic variants, including single nucleotide variations (SNVs) and small insertion/deletions, were called using Mutect2 implemented in GATK3.5. The resulting mutations were annotated using Variant Effect Predictor (v99) and CADD (v1.4, RRID:SCR_018393), including population frequency in control databases (gnomAD, RRID:SCR_014964). Whole-genome sequencing data was processed similar to whole exome data, with regards to preprocessing and alignment. To detect somatic structural variations, we used MANTA.

RNA-seq data were aligned using STAR (v2.4, RRID:SCR_004463), and transcripts were quantified using Kallisto (RRID:SCR_016582) based on the GENCODE v34 transcriptome. We summarized read count data using tximport R package, and then normalized read counts using regularized logarithm transformation (rlog) implemented in DESeq2. To determine cluster memberships, we used the ClusterR package to apply Gaussian mixture model clustering. Differential expression testing and gene co-expression network analysis were performed as described in the methods. Gene co-expression networks were constructed using the normalized lmQCM (local maximal Quasi-Clique Merger) framework, with minor modifications as noted in the methods. An adjacency matrix was obtained by calculating a robust correlation matrix using bicor function (biweight midcorrelation) Gene set overrepresentation analysis was performed using clusterProfiler and MSigDB (msigdb_v2022.1.Hs) for each module.

ChIP-seq reads were aligned using Bowtie 2 (RRID:SCR_005476). Peaks were identified using MACS2, and super-enhancers were detected using Rank Ordering of Super-Enhancers (ROSE, RRID:SCR_017390). For Hi-ChIP data, paired-end sequencing reads were processed using the Hi-C Pro pipeline, including alignment to hg19 using Bowtie 2. Topologically associated domains were inferred using SpectralTAD. Chromatin interaction loops were identified using FitHiChIP. To visualize the interaction matrix we used GENOVA R package.

For Hi-ChIP data, paired-end sequencing data were processed using the Hi-C Pro pipeline, including alignment to hg19 using Bowtie 2. For meningiomas without IHH (Indian Hedgehog) tandem duplications, data from three unique patients (MN-52407, MN-52430, and MN-60826) were combined, generating a total of 423.4 million paired reads. For meningiomas with IHH tandem duplications, data from 2 patients (MN-52323 and MN-61891) were combined, generating a total of 785.1 million paired reads. Fragments were mapped to MboI restriction sites using a digested reference genome generated by the 'digest_genome.py' script of Hi-C Pro. Iterative correction and eigenvector decomposition (ICE) normalization was performed on all datasets with a bin size (or resolution) of 50 kb. Topologically associated domains were inferred by using SpectralTAD. Chromatin interaction loops were identified using FitHiChIP at a 1% false discovery rate (FDR). We used H3K27ac ChIP-seq peak calls from MN-52323 and MN-52407 for IHH tandem duplication and non-IHH data, respectively, as input for FitHiChIP. We adopted a "peak to all" option so that only pairs of bins in which at least one bin overlapped H3K27ac peak. To visualize the interaction matrix (figure 5) we used GENOVA R package92.

For manuscripts utilizing custom algorithms or software that are central to the research but not yet described in published literature, software must be made available to editors and reviewers. We strongly encourage code deposition in a community repository (e.g. GitHub). See the Nature Portfolio guidelines for submitting code & software for further information.

## Data

Policy information about availability of data

All manuscripts must include a data availability statement. This statement should provide the following information, where applicable:

- Accession codes, unique identifiers, or web links for publicly available datasets
- A description of any restrictions on data availability
- For clinical datasets or third party data, please ensure that the statement adheres to our policy

The publicly available RNA-seq and ChIP-seq data used in this study are available in the Gene Expression Omnibus database under accession code GSE85135 (https://www.ncbi.nlm.nih.gov/geo/query/acc.cgi?acc=GSE85135) 14. The de-identified sequencing datasets generated as a part of this study, including RNA-sequencing, whole-exome sequencing (new drivers), H3K27ac ChIP-seq and H3K27ac Hi-ChIP, are deposited in the European Nucleotide Archive, under accession code PRJEB55424 (https://www.ebi.ac.uk/ena/browser/view/PRJEB55424). The remaining data are available within the Article, Supplementary Information, Supplementary Data or Source Data file.

## Human research participants

Policy information about studies involving human research participants and Sex and Gender in Research.

| | |
|---|---|
| Reporting on sex and gender | Our study population is a representative cross-section of mutation-negative meningiomas at several large academic medical centers. Sex and gender were not considered during study design because of the limited availability of samples. However, data on sex has been provided for samples in supplementary table 1. |
| Population characteristics | Population characteristics of the included patients are representative of the institutions in which samples were collected. The demographics of our cohort are consistent with previous studies of meningiomas, with enrichment of female and older patients. The average age among patients with available data is 58.1 years, and our cohort is 31.0% male. |
| Recruitment | Tumors were selected for analysis based on availability of frozen specimens. All patients gave informed consent for participation in research studies. |
| Ethics oversight | Institutional Review Board approval and written patient informed consents were obtained at all participating sites, including at Yale University (the principal site of this investigation), Bahcesehir University, Marmara University, University of Bonn, Hopital Pitie-Salpetriere, University Hospital of Cologne, University of Pittsburgh and Acibadem Mehmet Ali Aydınlar University. This study is in compliance with all relevant ethical regulations. |

Note that full information on the approval of the study protocol must also be provided in the manuscript.

# Field-specific reporting

Please select the one below that is the best fit for your research. If you are not sure, read the appropriate sections before making your selection.

☒ Life sciences ☐ Behavioural & social sciences ☐ Ecological, evolutionary & environmental sciences

For a reference copy of the document with all sections, see nature.com/documents/nr-reporting-summary-flat.pdf

# Life sciences study design

All studies must disclose on these points even when the disclosure is negative.

| | |
|---|---|
| Sample size | Sample size was determined based on the availability of specimens, with particular focus on cases that lacked known driver mutations. |
| Data exclusions | Samples were excluded during the driver discovery phase based on the quality of copy number data (as described in the methods). |
| Replication | Multiple samples from each molecular subgroup underwent gene expression analysis using RNA-seq, with consistent replication results (see figure 3a, supplemental figure 5). H3K27ac ChIP-seq replicates also exhibited consistent results (supplemental figure 11). Hi-ChIP experiments included two tandem-duplication replicates, and three wild-type replicates for comparison. All Hi-ChIP experiments were successful. |
| Randomization | Experimental groups are not reported in this study, which focuses on molecular characterization of meningiomas. |
| Blinding | RNA clustering results were obtained agnostic to driver event or other clinical features. Annotation of subgroup is performed after clustering. Subsequent analyses, including gene co-expression networks, were performed in a subgroup specific manner. Hi-ChIP experiments were designed specifically to compare meningiomas with and without tandem duplications on chromosome 2q35. Unless otherwise noted, all analyses have been performed uniformly for each data modality, without consideration of driver events or other molecular features. |

# Reporting for specific materials, systems and methods

We require information from authors about some types of materials, experimental systems and methods used in many studies. Here, indicate whether each material, system or method listed is relevant to your study. If you are not sure if a list item applies to your research, read the appropriate section before selecting a response.

## Materials & experimental systems

| n/a | Involved in the study |
|---|---|
| ☐ | ☒ Antibodies |
| ☒ | ☐ Eukaryotic cell lines |
| ☒ | ☐ Palaeontology and archaeology |
| ☒ | ☐ Animals and other organisms |
| ☒ | ☐ Clinical data |
| ☒ | ☐ Dual use research of concern |

## Methods

| n/a | Involved in the study |
|---|---|
| ☐ | ☒ ChIP-seq |
| ☒ | ☐ Flow cytometry |
| ☒ | ☐ MRI-based neuroimaging |

## Antibodies

| | |
|---|---|
| Antibodies used | H3K27ac antibody (Abcam ab4729, H3K27ac ChIP-seq and Hi-ChIP). Secondary Alexa fluorophore 555 (ThermoFisher Scientific) and Alexa fluorophore 647 (ThermoFisher Scientific). anti-SSTR2 (Ab134152; 1/2000; Rabbit). anti-GAB1 (Cell signaling Polyclonal 3232; 1/100; Rabbit) |
| Validation | All antibodies have been extensively validated by the manufacturers for the indicated modalities. Additional validation and optimization was performed on-site with each antibody for the species (human) and application of our study. |

## ChIP-seq

### Data deposition

☒ Confirm that both raw and final processed data have been deposited in a public database such as GEO.

☒ Confirm that you have deposited or provided access to graph files (e.g. BED files) for the called peaks.

| | |
|---|---|
| Data access links<br>*May remain private before publication.* | All ChIP-seq data generated in this study has been uploaded to ENA (accession PRJEB55424). Several additional samples were included in this analysis from GSE85135, as noted in Supplemental Table 1. |
| Files in database submission | Raw FASTQ files and BED peak files are provided. |
| Genome browser session<br>(e.g. UCSC) | https://genome.ucsc.edu/s/mark.youngblood/Enhancer%20Hijacking%20in%20Meningiomas |

### Methodology

| | |
|---|---|
| Replicates | Data from 37 ChIP-seq replicates are included in this study, including 20 samples that are previously reported. |
| Sequencing depth | Sequencing data was acquired on the Illumina HiSeq platform using single-end 75 base pair reads. A total of 30-50M reads was targeted for each sample. |
| Antibodies | H3K27ac antibody (Abcam ab4729, RRID:AB_2118291) |
| Peak calling parameters | ChIP enriched peaks (narrow peaks) were identified using MACS2, with these parameters: gsize = 2.7e9 qvalue = 0.05 |
| Data quality | Peaks were called using MACS2, with cutoff q value < 0.05. All data was manually inspected to ensure peak quality. |
| Software | Super-enhancers were detected using Rank Ordering of Super-Enhancers (ROSE, RRID:SCR_017390) with a slight modification for efficiency: (1) Peak stitching step was re-implemented in R following the same algorithm of ROSE; (2) Coverage calculation was performed using multiBamSummary implemented in deepTools (RRID:SCR_016366); (3) The total number of mapped reads was calculated using SAMtools flagstat for treatment and input samples, respectively; and (4) Super-enhancer detection was performed using R functions calculate_cutoff and numPts_below_line implemented in ROSE (R-script ROSE_callSuper.R). |
